# Urate as a CO_3_^•−^ Scavenger and Regulator of SOD-1 and OGG1 Enzymes: Insights from DFT, Molecular Docking, and Molecular Dynamics

**DOI:** 10.3390/antiox15060761

**Published:** 2026-06-16

**Authors:** Ana Amić, Žiko Milanović, Denisa Mastiľák Cagardová

**Affiliations:** 1 Department of Chemistry, Josip Juraj Strossmayer University of Osijek, Ulica cara Hadrijana 8A, 31000 Osijek, Croatia; 2 Department of Science, Institute for Information Technologies, University of Kragujevac, Liceja Kneževine Srbije 1A, 34000 Kragujevac, Serbia; ziko.milanovic@uni.kg.ac.rs; 3 Department of Chemical Physics, Institute of Physical Chemistry and Chemical Physics, Slovak University of Technology in Bratislava, Radlinského 9, SK-812 37 Bratislava, Slovakia; denisa.cagardova@stuba.sk

**Keywords:** carbonate radical anion, urate, explicit hydration, SET mechanism, SOD1, OGG1

## Abstract

The potency of urate, an abundant human plasma antioxidant, in preventing oxidative damage caused by the carbonate radical anion CO_3_^•−^, was studied using quantum chemical calculations. The influence of microhydration of CO_3_^•−^/CO_3_^2−^ and urate^−^/urate^•^ couples on the thermodynamic and kinetics of the one-electron oxidation process was investigated. Depending on the degree of microhydration, the estimated rate constant for one-electron transfer is in the range of 2.0–7.3 × 10^9^ M^−1^ s^−1^, in good agreement with the experimental value of 1.3 × 10^9^ M^−1^ s^−1^. Modeling using vertical detachment energy and electron affinity, the driving forces of single electron transfer revealed urate(H_2_O)_6_^−^ and CO_3_(H_2_O)_9_^•−^ clusters as the most likely existing species in water. Molecular docking revealed a favorable interaction of urate with the catalytic pocket of SOD1. Urate binds more strongly to the anionic active center of SOD1 than the reference inhibitor LSC-1, indicating its potency to prevent HCO_3_^−^-supported CO_3_^•−^ formation. In contrast, the known OGG1 inhibitor TH13264 shows substantially stronger binding than urate, indicating urate’s weaker affinity toward the DNA repair enzyme catalytic pocket. The molecular dynamics data indicate that urate binding does not destabilize either SOD1 or OGG1. In light of increasing evidence that the major source of oxidative stress could be CO_3_^•−^, rather than the commonly assumed hydroxyl radical HO^•^, the obtained results indicate the inherent ability of plasma to combat oxidative stress induced by this selective, milder oxidant. Such an ability with respect to the non-selective, highly reactive HO^•^ does not exist in vivo.

## 1. Introduction

Reactive oxygen and nitrogen species (ROS/RNS) overproduced by endogenous or exogenous stimuli are the major contributors to oxidative stress conditions [1]. The literature is continuously filled with theoretical discoveries of highly potent scavengers of ROS/RNS. Presumed scavengers are mainly natural phenolic compounds with very low concentration in biological sources of the human diet, usually much below 1 μM (and thus with poor bioaccessibility and bioavailability), and mostly with scarce water solubility. Consequently, their effective in vivo activity is questionable because physiological concentration could be a more important limitation than kinetics. Those facts have been reviewed and emphasized a decade ago [2], but are commonly overlooked or ignored by theoreticians. Earlier, radical scavenging was synonymous with antioxidant action. Free radicals were considered only as ‘bad gays’, and their elimination by scavenging seemed to be a key for suppressing oxidative stress [3]. Also, direct scavenging was designated as ‘primary’ antioxidant activity. Now, it is well-known that a plethora of antioxidant pathways exists, and their underlying mechanisms are far from completely elucidated, as well as the possible ranking of their in vivo physiological significance [4]. Moreover, enzyme reactions usually enable antioxidant defense rather than nonenzymatic direct radical scavenging [2].

On the other hand, despite the progress made, there are many challenges regarding activities and modes of action of existing low-molecular-weight antioxidants present in high concentration in the human body. One such compound is uric acid, the final product of purine metabolism. In aqueous environments, uric acid is present as urate (monovalent anion). The daily production and excretion of uric acid in males amounts to 600–700 mg [5]. The disruption of this homeostasis has been associated with the risk of gout and associated medical conditions such as cardiovascular diseases [6]. Normal urate concentrations in human plasma and serum are very high, 150–450 μM [7] and 155–430 μM [8], respectively, and somewhat lower in intracellular and other body fluids. Urate is the main contributor to the antioxidant capacity of human plasma (40–55%), overwhelming ascorbate (8–15%), α-tocopherol (<10%), and flavonoids (<2%) [7,9]. Thus, it is an important endogenous antioxidant capable of suppressing oxidative stress conditions by protecting the cell membrane, DNA, enzymes, and other biological molecules against oxidation by ROS/RNS. For example, experimental and theoretical evidence exist that uric acid can be efficient for repairing damaged protein residues [10,11,12].

One of the ROS is the carbonate radical anion, CO_3_^•−^, a species which attracts increased attention due to its presumed substantial involvement in oxidative stress genesis [13,14,15]. For years, it has been believed that the major cause of oxidative stress is the hydroxyl radical, HO^•^ [16]. This extremely reactive species non-selectively reacts at the place of its generation with any nearby biomolecule, mainly with diffusion-controlled rates [17]. Because of that, HO^•^-effective in vivo direct scavenging is impossible: there are no enzymatic systems and scavengers capable of eliminating it [2,18]. Increasing evidence suggests that at physiological conditions, CO_3_^•−^, not HO^•^, is the major source of oxidative stress because in vivo in the HCO_3_^−^/CO_3_^2−^ buffering system CO_3_^•−^ is produced, rather than HO^•^ [19]. CO_3_^•−^ is a powerful oxidant but milder than HO^•^: its reactions with various biological molecules are several orders of magnitude slower than the reactions of HO^•^ [20,21]. CO_3_^•−^ can diffuse away from the place of its generation and selectively oxidize critical biomolecules, e.g., amino acids, proteins, enzymes, lipids, and DNA. Unlike HO^•^, it could be in vivo eliminated by plasma antioxidants by direct scavenging.

When considering free radical scavenging as an antioxidant mechanism in physiological water environment, several common pathways could be operative: formal hydrogen atom transfer (fHAT), sequential proton loss followed by electron transfer (SPLET), single electron transfer (SET), single electron transfer followed by proton transfer (SET-PT), and radical adduct formation (RAF) [22]. Amongst them, it has been found that the SET is the most favorable mechanism for scavenging CO_3_^•−^. For example, SET from 2′-deoxyguanosine to CO_3_^•−^ proceeds faster than fHAT and RAF [23]. Also, SET as the operative pathway was considered in scavenging of CO_3_^•−^ by urate, ascorbate, and caffeate [24], α-tocopherol, α-CEHC, and gallic acid [25], and Trolox [26].

In water environments, both urate and CO_3_^•−^ are anticipated to be strongly solvated and can form the water clusters urate(H_2_O)*_n_*^−^ [27] and CO_3_(H_2_O)*_n_*^•−^ [28,29,30,31]. The formation of a hydration shell via hydrogen bonds with water molecules alters their molecular structure and physicochemical properties. The number of water molecules in urate(H_2_O)*_n_*^−^ and CO_3_(H_2_O)*_n_*^•−^ clusters has not been unequivocally defined so far.

The first goal of this research is to theoretically estimate the potency of urate for direct scavenging of CO_3_^•−^ under physiological conditions, as well as to determine the size of existing urate(H_2_O)*_n_*^−^ and CO_3_(H_2_O)*_n_*^•−^ clusters. This is achieved by examining the thermodynamics and kinetics underlying the SET mechanism and by analyzing the relationships between the VDE (vertical detachment energy) and AEA (adiabatic electron affinity), the main driving forces of electron transfer, and the reaction rate.

The second goal of this study is to ascertain the ability of urate to inhibit the peroxidase activity of the cytosolic isoform of superoxide dismutase (SOD) SOD1, i.e., Cu,Zn-SOD. SOD is known as an antioxidant enzyme that catalyzes the dismutation of O_2_^2−^ to generate H_2_O_2_ and O_2_, i.e., inhibits the damage triggered by O_2_^2−^. On the other hand, SOD-1 may act as a prooxidant [32]. Namely, at physiological conditions in the presence of H_2_O_2_ and HCO_3_^−^, SOD-1 peroxidase activity produces CO_3_^•−^. It is proposed that at the active site of SOD1, hydrogen peroxide forms a copper-bound oxidant [33] which oxidizes HCO_3_^−^, anchored at anionic binding site, to CO_3_^•−^ or inactivates the enzyme by oxidation of adjacent histidine residues [34]. CO_3_^•−^ is also capable of oxidizing histidine residues as well as diffusing out of the active site to induce oxidation of surrounding biomolecules. In vitro evidence suggests that urate may prevent H_2_O_2_-induced inactivation of SOD1, but the underlying mechanism is not fully elucidated [35].

Also, the potential of urate to inhibit or activate the DNA repair enzyme 8-oxoguanine DNA glycosylase 1 (OGG1) was investigated. OGG1 is a promising multifunctional therapeutic target for the treatment of oxidative DNA damage-related diseases, such as malignant tumors, age-related disorders, and incurable inflammation [36].

## 2. Materials and Methods

### 2.1. DFT Computation

The density functional theory (DFT) represents a powerful tool for studying free radical scavenging mechanisms of natural compounds [22]. The M06-2X functional and 6–311++G(d,p) basis set was used to perform the optimization and frequency calculation of urate and CO_3_^•−^ species involved in the SET mechanism. The M06-2X functional has been chosen because it is designed for main-group chemistry and recommended for thermochemical kinetics [37]. It has been recognized as particularly suitable for the estimation of thermodynamics and kinetics of reactions involving free radicals [22]. Explicitly hydrated reactants are immersed in the solvent continuum modeled by the SMD solvation approach [38]. For open-shell systems, unrestricted calculations were performed. The spin operator 〈*S*^2^〉 values for radical species, before and after the annihilation of the first spin contaminant, have been checked to avoid possible untrue results. Spin contamination can be considered negligible if the value of 〈*S*^2^〉 differs from the correct value (〈*S*^2^〉 = 0.75 for a pure doublet) by less than 10%. All computations were performed using the Gaussian 09 program package [39].

### 2.2. Estimations of Thermodynamic and Kinetic Data

The Eyringpy program [40] was used for calculations of thermodynamic and kinetic data. The Gibbs free energy of activation, ${\Delta G}_{SET}^{\neq }$, of investigated SET reactions was estimated by using Marcus theory [41]. This theory is based on the transition state theory (TST) and enables calculating the barrier of any SET reaction from two thermodynamic parameters: the free energy of the reaction, ${\Delta G}_{SET}^{0}$, and the nuclear reorganization energy, λ:

(1) $${\Delta G}_{SET}^{\neq }=\frac{\lambda }{4}{\left(1+\frac{{\Delta G}_{SET}^{0}}{\lambda }\right)}^{2}$$

(2) $$\lambda \;\approx \;{\Delta E}_{SET}-{\Delta G}_{SET}^{0}$$

${\Delta E}_{SET}$ is the nonadiabatic energy difference between reactants and vertical products for SET.

The TST rate constant for the SET reaction, *k*^TST^ is calculated as

(3) $$k^{\mathrm{TST}}=\frac{k_{B}T}{h}\;e^{-({\Delta G}_{SET}^{\neq })/RT}$$

*k*_B_ is the Boltzmann constant, *T* is the temperature, *h* is the Planck constant, and *R* is the gas constant.

If *k*^TST^ is close to the diffusion limit (*k*^TST^ > 10^9^ M^−1^ s^−1^), the apparent rate constant, *k*_app_, and rate constant for an irreversible bimolecular diffusion-controlled reaction, *k*_D_, were calculated:

(4) $$k_{\mathrm{app}}\;=\;\frac{k_{D}\;k_{TST}}{k_{D}\;+\;k_{TST}}$$

*k*_D_ = 4π*R*_AB_*D*_AB_*N*_A_ (5)

*R*_AB_ is the reaction distance, and *D*_AB_ is the mutual diffusion coefficient of the reactants A and B, and *N*_A_ is the Avogadro constant.

The rate constant $k_{Mf}^{SET}$ for the SET mechanism at pH 7.4 was calculated involving *k*_app_ and the molar fraction of both reactants, i.e., ^M^*f*_urate_ = 0.9862 for urate, and ^M^*f*_CO3•−_ = 1 for carbonate radical anion:

(6) kMfSET=kapp×furateM×fCO3•−M

Molar fractions were estimated from experimentally determined p*K*_a_ values. ^M^*f*_CO3•−_ amounts to 1 because CO_3_^•−^ is a conjugated base of a strong acid, HCO_3_^•^ (p*K*_a_ < 0 [42]). Such a calculated rate constant $k_{Mf}^{SET}$ is directly related to the experimentally determined one under the same conditions.

The vertical detachment energy (VDE) is defined as the difference in electronic energy of urate and its corresponding radical, both in the optimized geometry of urate [43].

The electron affinity (EA) is the difference in electronic energy of CO_3_^•−^–water cluster and CO_3_^2−^–water cluster in their respective optimized geometry [44].

### 2.3. Molecular Docking and Binding Affinity Evaluation

To explore the interaction profile and binding affinity of urate toward SOD1 and OGG1, a detailed molecular docking analysis was performed using AutoDock Tools 1.5.7 in combination with AutoDock 4.2.6 [45]. A structured and validated docking protocol was applied to ensure the reliability and reproducibility of the obtained results [46,47,48]. The urate structure was reoptimized at the B3LYP-D3BJ/6-311+G(d,p) level of theory, providing an accurate description of the ligand’s geometrical and electronic properties [49,50]. The crystal structures of SOD1 (PDB ID: 1CB4) and OGG1 (PDB ID: 8BVX) were retrieved from the Protein Data Bank [51]. Protein preparation was conducted using BIOVIA Discovery Studio 2021 [52], including the removal of co-crystallized ligands, water molecules, and non-essential heteroatoms to avoid artificial interactions during docking simulations. For SOD1, a grid box of 40 × 40 × 40 Å was centered at coordinates (10.41, 87.88, 18.62 Å) in order to encompass the known catalytic/electrostatic channel of SOD1, located in the vicinity of the Cu/Zn metal binding region and amino acid residues relevant for substrate access and stabilization, whereas for OGG1, a grid box of 29 × 29 × 29 Å was defined with the grid center positioned at (−8.70, −15.91, 12.50 Å), based on the selected binding regions. Docking calculations were performed using the Lamarckian Genetic Algorithm (LGA), which enables efficient sampling of flexible ligand conformations within a rigid protein framework [53]. The following docking parameters were applied: population size of 150, maximum number of 2,500,000 energy evaluations, 27,000 generations, mutation rate of 0.02, and crossover rate of 0.8. For each protein–ligand system, 100 independent docking runs were carried out to ensure statistically significant sampling and identification of the most probable binding conformations.

### 2.4. Molecular Dynamics Study

To evaluate the structural stability and interaction dynamics of the urate-SOD1 and urate–OGG1 complexes, molecular dynamics (MD) simulations were performed using the AMBER 22 simulation package [54]. The ff14SB force field was employed for the protein component. In contrast, ligand parameters and system preparation were generated through the CHARMM-GUI platform, enabling the construction of AMBER-compatible topology and coordinate files [55,56]. The Cu and Zn ions were retained and treated using standard non-bonded ion parameters from the CHARMM toppar libraries, without additional bonded or QM/MM treatment of the metal coordination sphere. The docked complexes exhibiting the most favorable binding modes were embedded in a truncated octahedral periodic box filled with explicit TIP3P water molecules, maintaining a minimum distance of 10 Å between the solute and the box boundary [57]. System neutrality and physiological ionic strength (0.15 M) were achieved by introducing appropriate numbers of K^+^ and Cl^−^ counterions. Prior to the production phase, a two-step energy minimization protocol was applied. Initially, solvent molecules and ions were relaxed while positional restraints were maintained on the solute (2500 steps of steepest descent followed by 2500 steps of conjugate gradient). Subsequently, the entire system was subjected to unrestrained minimization. Thermal equilibration was then carried out in sequential stages: a 1 ns NVT simulation at 300 K using the Langevin thermostat (collision frequency of 2 ps^−1^), followed by a 1 ns NPT equilibration at 1 atm with pressure control implemented via the Berendsen barostat [58]. Covalent bonds involving hydrogen atoms were constrained using the SHAKE algorithm, allowing an integration time step of 2 fs [59]. The final production simulation was conducted for 150 ns under NPT conditions, and trajectory coordinates were recorded every 10 ps for subsequent structural and energetic analyses.

## 3. Results and Discussion

**Figure 1:**
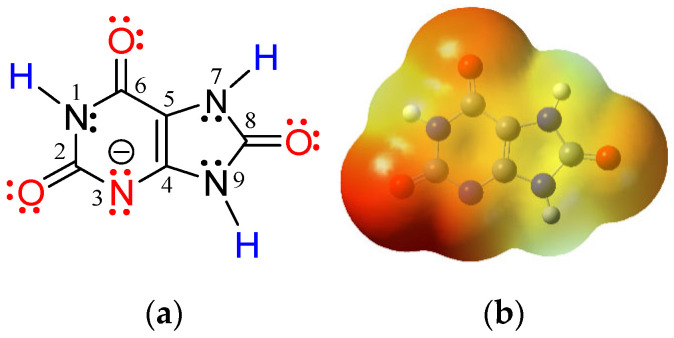
(**a**) Lewis structure and atom numbering in urate. H-bond donor sites (in blue) and H-bond acceptor sites (in red) are assigned. (**b**) Electron density map of urate. The red regions indicate the negative area.

In neutral aqueous environments, uric acid is present almost completely as urate, a monoanion species formed by deprotonation of the imino-hydrogen atom in position 3-N [60]. Lewis structure of urate ascribes a negative charge to the 3-N atom (Figure 1a), but a more reliable picture arises from the electron density map of urate [61]: the distribution of negative charge is mainly located on carbonyl oxygens (Figure 1b).

Experimentally determined p*K*_a_ values of uric acid amount to 5.4 and 9.8 [62], which implies that at a physiological pH of 7.4, molar fractions of uric acid, monovalent anion (urate), and divalent anion are 0.009862, 0.9862, and 0.003926, respectively. Because the electron-donating/accepting ability of urate(H_2_O)*_n_*^−^ and CO_3_(H_2_O)*_n_*^•−^ clusters undoubtedly depends on their geometry, minimal energy clusters are required for the estimation of thermodynamics and kinetics of the underlying SET mechanism.

### 3.1. Conformational Analysis

Urate has seven hydrogen bond binding sites available to chelate water molecules: oxygens of three carbonyl groups and nitrogen of the deprotonated imino group may act as a H-atom acceptor, while three imino groups may act as a H-atom donor (Figure 1a). On the other hand, each water molecule may form up to four H-bonds, two via donation of H-atoms and two via the H-atom accepting oxygen atom. For uric acid, it has been shown that water molecules are H-bonded to adjacent oxygen and hydrogen atoms (as well as between themselves), rather than to an isolated oxygen or hydrogen [27]. It could be expected that water molecules in the first hydration shell of urate follow this arrangement. Undoubtedly, the H-bond network is dynamic and alternates due to thermal excitation: H-bonds may be broken and rearranged in a picosecond to nanosecond time scale [63].

Based on the above-mentioned results of Cai et al. [27] twenty initial guess structures for the urate(H_2_O)_3_^−^ cluster and thirty for the urate(H_2_O)_6_^−^ cluster were generated, and full geometry optimizations and frequency calculations were performed. The hydration energy (*E*^hydr^) of all clusters was calculated by using Equation (7) [64]:

*E*^hydr^ = *E*_urate(H2O)*n*−_ − (*nE*_H2O_ + *E*_urate−_) (7)

where *E*_urate(H2O)*n*−_, *E*_H2O_, and *E*_urate−_ are the total energy of the cluster, the single water molecule, and the free urate, respectively. *E*^hydr^ relates to the interaction energy of urate with its hydrated shell of *n* H_2_O molecules and increases with the increase of the number of H-bonded H_2_O molecules [27]. The negative sign of *E*^hydr^ indicates spontaneity of urate hydration and the dominant conformer possesses the lowest *E*^hydr^. Equilibrium population (in %) was predicted by the Boltzmann distribution based on the total energy (*E* in *a.u*.) of all conformers. Five minimal energy cluster structures of urate hydrated by 3 and 6 water molecules are presented in Table 1. Complete results are presented in Tables S1 and S2 in Supplementary Material.

Revealed minimal energy conformer of urate(H_2_O)_3_^−^ cluster and urate(H_2_O)_6_^−^ cluster, along with free and fully hydrated urate, were used in the estimation of their scavenging potency. Their Cartesian coordinates are given in Tables S3 and S4.

Comprehensive conformational analysis of CO_3_(H_2_O)*_n_*^•−^ clusters, including *n* = 1–8, has been performed by Pathak et al. [28]. We used global minimum energy structures, i.e., conformers for *n* = 4 and 6 from this work. Dooley and Vyas [31] used 0 to 30 explicit water molecules and different DFT functionals to reproduce the aqueous reduction potential of CO_3_^•−^. Minimum energy clusters of CO_3_(H_2_O)*_n_*^•−^ (*n* = 9, 12, 15, and 24) from this work were taken. All used conformers were reoptimized at the applied SMD/M06-2X/6-311++G(d,p) level of theory. Corresponding Cartesian coordinates are given in Tables S5–S8. Conformer with *n* = 6 was used by Hebert and Schlegel [30] in a study of oxidation of guanine. Conformers with *n* = 6 and *n* = 9 were recently used by us in the estimation of antiradical potency of four phenolic antioxidants [25,26].

As already noted, the spin operator 〈*S*^2^〉 values for all of the urate and CO_3_^•−^ radical species have been checked. In all cases, the deviations before and after annihilation of the first spin contaminant from the ideal value for a pure doublet (〈*S*^2^〉 = 0.75) were lower than 1.60% and 0.01%, indicating that the obtained energy values of radical species studied in this work are reliable (Table S9).

### 3.2. Estimation of Urate(H_2_O)_n_^−^ Cluster Potency in the Scavenging of the CO_3_(H_2_O)_n_^•−^ Cluster

The obtained results for SET from urate(H_2_O)*_n_*^−^ clusters (*n* = 0, 3, 6 and 10) to CO_3_(H_2_O)*_n_*^•−^ clusters (*n* = 0, 4, 6, 9, 12, 15 and 24) in water at pH = 7.4 are presented in Table 2 (selected data) and in Tables S9–S12 (complete data). Altogether 28 reactions are modeled to ascertain the potency of urate to inactivate CO_3_^•−^ at physiological conditions, as well as to predict the most reliable size of reactants. All modeled reactions are thermodynamically feasible (Δ_r_*G* < 0), except the reaction of fully hydrated urate with free CO_3_^•−^, and diffusion-controlled *k*^TST^ > 10^9^ M^−1^ s^−1^, i.e., in the range of 2.7 × 10^9^ M^−1^ s^−1^ to 6.2 × 10^12^ M^−1^ s^−1^. In accordance with the Evans–Polanyi principle [65], for the reaction of CO_3_(H_2_O)*_n_*^•−^ clusters (*n* = 0, 4, 6, and 9) with all urate(H_2_O)*_n_*^−^ clusters, the logarithms of the *k*^TST^ constants are inversely fully correlated to the Δ_r_*G* (*r* ≥ −0.99), Figure S1. However, if the number of H_2_O molecules in the CO_3_(H_2_O)*_n_*^•−^ cluster was enlarged to 12, 15, and 24, a linear trend was interrupted. The calculated AEA for CO_3_(H_2_O)*_n_*^•−^ clusters indicate that increased hydration produces stronger oxidizing agents. On the other hand, VDE of urate(H_2_O)*_n_*^−^ clusters increases with the number of hydrated water molecules, indicating less reactivity of more hydrated clusters.

The data presented in Table 2 show that an increased number of explicit water molecules in the CO_3_^•−^ hydration shell increases exergonicity, decreases reaction barrier height, and increases the TST rate constant of the SET mechanism, *k*^TST^. Because all *k*^TST^ are larger than the diffusion rate, they lack physical meaning [22]. To be comparable with experimentally determined rate constant, *k*^TST^ should be corrected by diffusion-controlled rate *k*_D_, thus giving apparent rate constant *k*_app_ (Tables S9–S12). Multiplying *k*_app_ with the molar fractions of reactants at a given pH produces the rate constant $k_{Mf}^{SET}$ which should match the assayed one. All predicted $k_{Mf}^{SET}$ are nearly equal due to the levelling effect of *k*_D_ on *k*_app_. $k_{Mf}^{SET}$ is in the narrow range of 2.0–7.6 × 10^9^ M^−1^ s^−1^ (Table 2) and reveals the high potency of urate to scavenge CO_3_^•−^. Obtained result is in good agreement with the experimentally determined value at pH = 7.4, which amounts to 1.3 (±0.4) × 10^9^ M^−1^ s^−1^ [66].

Electron transfer from urate to CO_3_^•−^ is mainly driven by the electron donating ability of urate and the electron accepting ability of CO_3_^•−^ [24]. Appropriate indices of those effects are VDE and AEA, respectively: lower VDE of urate clusters and higher AEA of CO_3_^•−^ clusters favor electron transfer. The question that arises is which number of explicit water molecules in CO_3_^•−^ and urate clusters satisfy reliable prediction of reaction kinetics. For example, Zilberg et al. [29] and Hebert and Schlegel [30] suggested that in aqueous solution CO_3_(H_2_O)_6_^•−^ cluster exists, while Dooley and Vyas [31] accurately predicted the reduction potential of CO_3_^•−^ by using the CO_3_(H_2_O)_9_^•−^ cluster.

**Figure 2:**
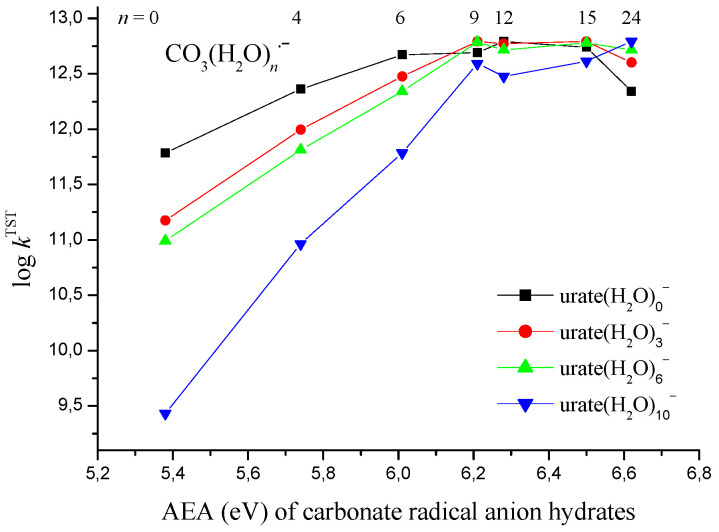
Plot of adiabatic electron affinity (AEA in eV) of CO_3_(H_2_O)*_n_*^•−^ clusters vs. logarithm of transition state theory rate constant (log *k*^TST^) for SET reaction of urate(H_2_O)*_n_*^−^ clusters with CO_3_(H_2_O)*_n_*^•−^ clusters.

To determine the number of explicit water molecules in the CO_3_^•−^ cluster, we analyzed the relationship between the AEA of the CO_3_^•−^ clusters and the reaction rate, i.e., log *k*^TST^. AEA of CO_3_^•−^ hydrated by 0, 4, 6, and 9 water molecules amounts to 5.38, 5.74, 6.01, and 6.21 eV, respectively, i.e., an increased number of water molecules in the first hydration shell facilitates the electron-accepting ability of CO_3_^•−^. Strong correlation exists between those AEAs and log *k*^TST^: correlation coefficient *r* amounts to 0.966, 0.997, 0.999, and 0.998 for the reaction of CO_3_^•−^ clusters with urate hydrated by 0, 3, 6, and 10 explicit water molecules, respectively. Results presented in Figure 2 and Table S13 showed that an increased number of explicit water molecules in the hydration shell of CO_3_^•−^ increases AEA, i.e., enables a faster reaction rate, while the increased microhydration of urate decreases reaction rate with CO_3_^•−^ clusters holding 0, 4, 6, and 9 water molecules. For any urate cluster (Tables S9–S12), CO_3_^•−^ hydrated by 9 water molecules produces nearly equal reaction rate, i.e., 9 explicit water molecules lead to a convergence of log *k*^TST^. An additional increase in CO_3_^•−^ hydration with 12, 15, and 24 water molecules has no significant impact on the reaction rate.

**Figure 3:**
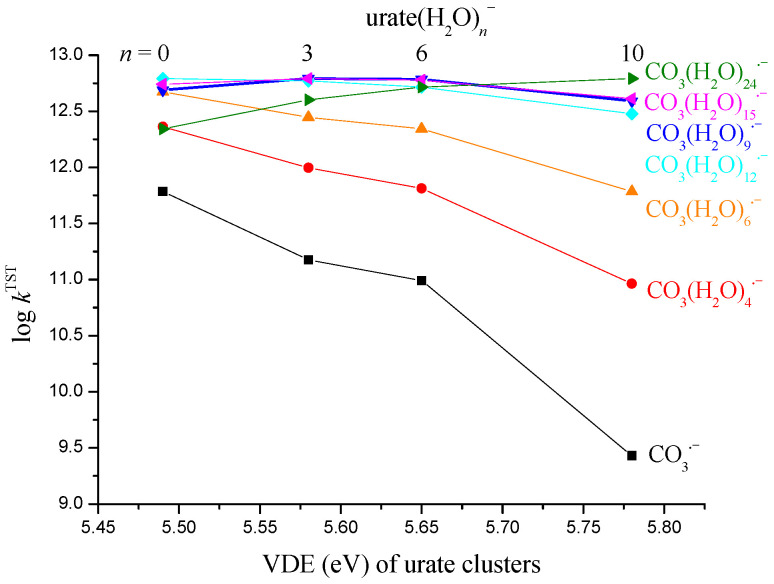
Plot of vertical detachment energy (VDE in eV) of urate clusters vs. logarithm of rate constant (log *k*^TST^) for SET reaction of urate(H_2_O)*_n_*^−^ clusters with CO_3_(H_2_O)*_n_*^•−^ clusters.

On the other hand, the VDE may serve as an index to ascertain the number of water molecules in the urate cluster, which leads to a convergence of reaction rate. The VDE of urate clusters increases with the increased number of explicit water molecules. For urate hydrated by 0, 3, 6, and 10 water molecules, VDE amounts to 5.49, 5.58, 5.65, and 5.78 eV, respectively. As already noted, the lower VDE enables a faster reaction rate. As Figure 3 and Table S14 show, this is valid for the reaction of urate clusters with CO_3_^•−^ hydrated by 0, 4, and 6 water molecules. Strong correlation exists between VDE and log *k*^TST^: *r* amounts to −0.971, −0.984, and −0.980. CO_3_^•−^ clusters with 9 and more water molecules lack such a relationship (except for *n* = 12), and all react with urate(H_2_O)_6_^−^ cluster by nearly equal rate, i.e., log *k*^TST^ ≈ 12.7. Thus, it appears that urate chelated by six water molecules is the most probable cluster in a water environment. The reaction of urate(H_2_O)_6_^−^ cluster with CO_3_(H_2_O)_9_^•−^ cluster proceeds with *k*^TST^ = 6.1 × 10^12^ M^−1^ s^−1^ (log *k*^TST^ = 12.785), i.e., corresponds to the result obtained by using AEA for estimating the most probable CO_3_^•−^–water cluster.

For urate(H_2_O)*_n_*^−^ and CO_3_(H_2_O)*_n_*^•−^ clusters, both vertical and adiabatic values of detachment energy and electron affinity were calculated (Table 2). The correlation between VDE and ADE of urate(H_2_O)*_n_*^−^ clusters amounts to *r* = 0.985, and between VEA and AEA of CO_3_(H_2_O)*_n_*^•−^ clusters amounts to *r* = 0.971. This indicates that the geometric relaxation of the studied species does not significantly modify the trends observed from the vertical values.

In summary, the results presented in this section suggest that at physiological pH = 7.4 both urate and CO_3_^•−^ exist as hydrated species: performed analysis reveals urate(H_2_O)_6_^−^ and CO_3_(H_2_O)_9_^•−^ clusters as the most probable amongst eleven investigated. SET reaction between them is diffusion-controlled and proceeds with estimated $k_{Mf}^{SET}$ rate constant of 7.3 × 10^9^ M^−1^ s^−1^, Table 2. The potency of urate as CO_3_^•−^ scavenger is similar to that predicted for α-CEHC ($k_{Mf}^{SET}$ = 7.5 × 10^9^ M^−1^ s^−1^ [25]), and Trolox ($k_{Mf}^{SET}$ = 7.5 × 10^9^ M^−1^ s^−1^ [26]), and higher than that of α-tocopherol ($k_{Mf}^{SET}$ = 1.5 × 10^7^ M^−1^ s^−1^ [25]) and gallate ($k_{Mf}^{SET}$ = 3.5 × 10^8^ M^−1f^ s^−1^ [25]). However, α-CEHC has a low concentration in plasma (<1 μM [67]), indicating its reduced potency in CO_3_^•−^ scavenging, while Trolox is a synthetic water-soluble analogue of vitamin E.

### 3.3. Molecular Docking Study of Urate in Different Positions of the SOD1 and OGG1 Proteins

The molecular docking results indicate that urate interacts with both investigated enzymes, SOD1 and OGG1, with moderate binding affinity (Table 3). For SOD1, the calculated binding free energy is −5.25 kcal mol^−1^, corresponding to an inhibition constant of 0.14 mM. The binding is predominantly stabilized by intermolecular interactions (Δ*G*_inter_ = −5.25 kcal mol^−1^), mainly originating from van der Waals, hydrogen bonding, and desolvation contributions (−5.13 kcal mol^−1^), while the electrostatic component is relatively small (−0.12 kcal mol^−1^).

A somewhat weaker interaction is observed for OGG1, where urate exhibits a binding free energy of −3.99 kcal mol^−1^ with a calculated inhibition constant of 1.19 mM. Similarly to the SOD1 system, stabilization is largely governed by dispersion and hydrogen bonding interactions (−3.94 kcal mol^−1^), whereas electrostatic contributions remain minimal (−0.05 kcal mol^−1^).

Comparison with reference inhibitors reveals that urate binds more strongly to SOD1 than the standard inhibitor LSC-1 (Δ*G*_bind_ = −3.62 kcal mol^−1^), suggesting a relatively favorable interaction within the SOD1 active site. In contrast, the known OGG1 inhibitor TH13264 shows substantially stronger binding (Δ*G*_bind_ = −7.44 kcal mol^−1^) than urate, indicating that the investigated ligand exhibits weaker affinity toward the OGG1 catalytic pocket.

**Figure 4:**
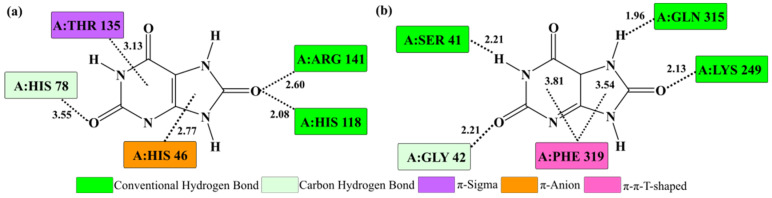
Two-dimensional map of key intermolecular interactions between urate and amino acid residues in the active sites of SOD1 (**a**) and OGG1 (**b**) obtained from molecular docking analysis. Interatomic distances are given in Å, while colors indicate different types of noncovalent interactions.

The analysis of intermolecular interactions indicates that urate establishes several stabilizing contacts within the active sites of both enzymes (Figure 4). In the SOD1 active site, the ligand forms multiple hydrogen bonds with key amino acid residues, including ARG 141 (2.60 Å) and HIS 118 (2.08 Å), which play an important role in positioning the ligand within the catalytic pocket. Additional interactions, such as a π–anion interaction with HIS 46 (2.77 Å) and a carbon–hydrogen bond with HIS 78 (3.55 Å), further stabilize the ligand orientation. Moreover, a π–σ interaction with THR 135 was observed, indicating that, in addition to hydrogen bonding, aromatic interactions also contribute to the stabilization of the complex.

As noted in the Introduction section, SOD1 can be a source of oxidative damage via peroxidase activity. By using H_2_O_2_ as a substrate, the enzyme–copper-bound oxidant (usually designated as Cu^2+^- ^•^OH) is formed at the active site of SOD1, which may inactivate the enzyme by damaging nearby histidine residues. In vitro experiments revealed that physiological levels of urate completely prevented the inactivation of SOD1. It has been suggested that urate restores the dismutase activity of SOD1 by the inactivation of Cu^2+^- ^•^OH at the active site of enzyme, accompanied by urate radical formation [34,35].

On the other hand, HCO_3_^−^ enhances SOD1 peroxidase activity via the production of CO_3_^•−^ [68]. HCO_3_^−^ is present in mM concentrations in cells and tissues and could be anchored at the anion-binding site of SOD1 (ARG 141) adjacent to the active site, enhancing oxidative damage via CO_3_^•−^ production [69,70,71]. Our results suggest that urate may establish transient interactions with ARG 141, which could potentially influence HCO_3_^−^ binding and CO_3_^•−^ formation, and this will be further investigated through molecular dynamics simulations. Thus, urate has a twofold role in suppressing peroxidase activity of SOD1: it may neutralize bound oxidant at the enzyme active site, and suppress HCO_3_^−^ dependent CO_3_^•−^ production by occupying anion-binding site of the enzyme.

In the OGG1 active site, urate forms hydrogen bonds with residues SER 41 (2.21 Å), GLN 315 (1.96 Å), and LYS 249 (2.13 Å), creating a network of polar contacts that stabilize the ligand within the binding pocket. In addition, a π–π T-shaped interaction with PHE 319 further contributes to the stabilization of the complex.

Overall, the interaction pattern suggests that hydrogen bonding represents the dominant stabilization factor in both enzyme–ligand complexes, while additional π interactions further contribute to the stability and proper accommodation of the ligand within the binding pockets of the enzymes.

### 3.4. Molecular Dynamics Study of Urate in Different Positions of the SOD1 and OGG1 Proteins

Molecular dynamics simulations were conducted to evaluate the structural stability and conformational behavior of SOD1 and OGG1 in their apo forms and in complex with urate. The obtained Root Mean Square Deviation (RMSD), Radius of Gyration (Rg), and Root Mean Square Fluctuation (RMSF) parameters collectively provide insight into global stability, compactness, and local flexibility of both systems.

**Figure 5:**
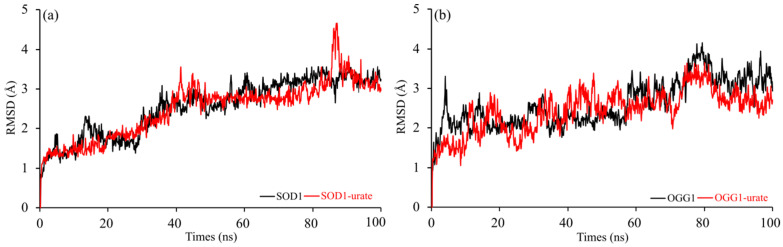
Comparative analysis of 100 ns molecular dynamics (MD) simulations showing RMSD (root mean square deviation) of the C–Cα–N backbone for the SOD1 (**a**) and OGG1 (**b**) enzyme with and without urate in active sites.

The backbone RMSD values indicate that both proteins remain structurally stable throughout the simulation, regardless of ligand presence (Figure 5). For SOD1, the average RMSD slightly decreased from 2.55 ± 0.70 Å in the apo form to 2.50 ± 0.71 Å in the urate-bound system. This minimal reduction, accompanied by nearly identical standard deviations, suggests that urate binding does not induce significant conformational perturbations. Instead, the structural fold of SOD1 is preserved, indicating that the ligand is accommodated without destabilizing the protein scaffold. A comparable trend was observed for OGG1, where RMSD decreased from 2.61 ± 0.58 Å in the free state to 2.44 ± 0.53 Å in complex with urate. The reduction in both the mean deviation and fluctuation amplitude suggests a modest stabilizing effect of urate on the OGG1 backbone dynamics. RMSD values within the range of approximately 2.4–2.6 Å further confirm that both systems reached equilibrium and maintained conformational consistency during the trajectory.

**Figure 6:**
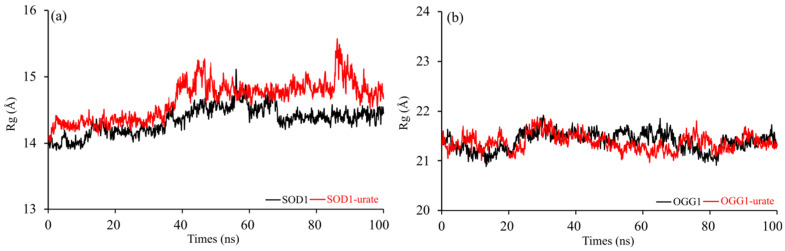
Comparative analysis of 100 ns molecular dynamics (MD) simulations showing Rg (radius of gyration) of the C–Cα–N backbone for the SOD1 (**a**) and OGG1 (**b**) enzyme with and without urate in active sites.

The radius of gyration analysis supports these observations by revealing only subtle differences in overall compactness (Figure 6). In the case of SOD1, Rg increased from 14.35 ± 0.20 Å to 14.65 ± 0.29 Å upon ligand binding. This slight expansion may reflect minor rearrangements of surface loops or adaptive breathing motions rather than large-scale conformational transitions. Importantly, the magnitude of change remains small, indicating preserved structural integrity. For OGG1, Rg values were nearly identical in the free (21.40 ± 0.19 Å) and bound (21.37 ± 0.16 Å) forms, suggesting that urate does not affect the global compactness of the enzyme. The consistency of Rg values reinforces the conclusion that no unfolding or structural collapse occurs in the presence of the ligand.

**Figure 7:**
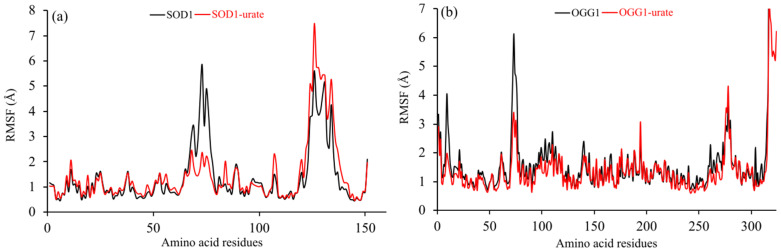
Comparative analysis of 100 ns molecular dynamics (MD) simulations showing RMSF (root mean square fluctuation) of the C–Cα–N backbone residues of SOD1 (**a**) and OGG1 (**b**) with and without urate in the active sites.

Residue-level flexibility, evaluated through RMSF analysis, reveals subtle differences between the two proteins (Figure 7). SOD1 exhibited a slight increase in average RMSF from 1.38 ± 1.14 Å to 1.47 ± 1.22 Å upon urate binding, indicating a marginal rise in local fluctuations. Such behavior may be attributed to adaptive adjustments in flexible surface regions while maintaining a stable core structure. In contrast, OGG1 displayed a decrease in average RMSF from 1.77 ± 1.62 Å in the apo state to 1.56 ± 1.21 Å in the complex, suggesting that ligand binding dampens local motions. This reduction in flexibility, together with the observed decrease in RMSD, implies a stabilizing interaction that restricts excessive backbone movement, particularly in regions proximal to the binding site.

Additionally, to further examine the proposed interaction between urate and the ARG 141 region of SOD1, hydrogen-bond occupancy, contact persistence, and distance analyses were performed along the MD trajectory. The obtained results showed that the urate–ARG 141 interaction was predominantly transient in nature, with low hydrogen-bond occupancy values and gradual increase of the intermolecular distance during the simulation. Contact analysis indicated that interactions between urate and ARG 141 were mainly present during the early stages of the trajectory, while becoming significantly less frequent in later simulation periods. These findings suggest that urate may transiently access the ARG 141 region within the SOD1 catalytic channel; however, the interaction does not appear sufficiently persistent to support stable competition with HCO3− under the simulated conditions.

Taken together, the molecular dynamics data indicate that urate binding does not destabilize either SOD1 or OGG1. Structural deviations remain within a stable range, global compactness is preserved, and local fluctuations show only moderate adjustments. While SOD1 undergoes a slight expansion accompanied by marginally increased flexibility, OGG1 demonstrates reduced backbone deviation and dampened residue-level mobility, suggesting a more pronounced stabilizing effect in the latter case. The MD results suggest that urate forms stable complexes with both enzymes without inducing structural destabilization. The observed reduction of RMSD and RMSF values in the OGG1 system indicates partial rigidification of the protein backbone, particularly near the binding region, which may be associated with inhibitory behavior.

## 4. Conclusions

The results of this study highlight the multifunctional antioxidant potential of urate by exploring its ability to directly scavenge CO_3_^•−^ and interact with both CO_3_^•−^-generating and DNA repair enzymes. This topic is of particular interest because of recent evidence that, under physiological conditions, instead of the commonly assumed HO^•^, CO_3_^•−^ could be the major source of oxidative damage. The reaction kinetics of CO_3_^•−^ scavenging by urate, an abundant plasma antioxidant, were estimated by using explicitly hydrated reactants immersed in a water continuum modeled by the SMD approach. Both urate and CO_3_^•−^ in an aqueous environment tend to be hydrated. The number of explicit water molecules in their first hydration shell is determined by analyzing the relationship between log *k*^TST^ and VDE and AEA, respectively. It was found that the urate(H_2_O)_6_^−^ cluster and the CO_3_(H_2_O)_9_^•−^ cluster are existing species at physiological pH 7.4. Obtained results indicate urate as a potent in vivo scavenger of CO_3_^•−^, more potent than α-tocopherol, α-CEHC, gallate, and Trolox. Molecular docking results indicate that urate can establish favorable interactions with the ARG 141 region within the catalytic channel of SOD1, while molecular dynamics simulations suggest that these interactions are predominantly transient in nature. Although the obtained computational results do not confirm definitive competition with HCO_3_^−^, they indicate a possible modulatory effect of urate on HCO_3_^−^-associated CO_3_^•−^ formation. In contrast, urate exhibits weaker binding toward OGG1 and does not impair its catalytic function. Molecular dynamics simulations further confirm the structural stability of both SOD1 and OGG1 in their apo forms and in complex with urate, indicating that ligand binding does not induce destabilization of either enzyme. Overall, these findings provide mechanistic insight into the dual antioxidant role of urate, combining efficient radical scavenging with modulation of enzyme-mediated oxidative pathways.

## Figures and Tables

**Table 1:** The fully optimized minimal energy structures of: (**a**) urate(H_2_O)_3_^−^ clusters, and (**b**) urate(H_2_O)_6_^−^ clusters, calculated at SMD/M06-2X/6-311++G(d,p) level of theory. Hydration energy *E*^hydr^ (kcal mol^−1^), the total energy of the cluster *E* (*a.u*.), and Boltzmann population (%).

(**a**)	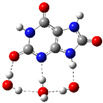	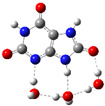	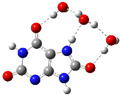	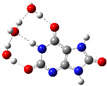	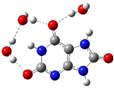
*E*^hydr^ (kcal mol^−1^)	−17.55	−17.46	−17.39	−16.97	−16.82
*E* (*a.u*.)	−866.55542497	−866.55527264	−866.55515990	−866.55449004	−866.55426240
population (%)	26.52	22.53	19.97	9.75	7.64
(**b**)	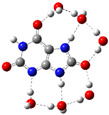	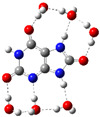	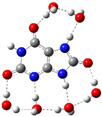	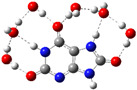	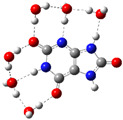
*E^hydr^* (kcal mol^−1^)	−35.10	−35.04	−34.86	−34.62	−34.39
*E* (*a.u*.)	−1095.88761877	−1095.88751127	−1095.88723963	−1095.88685498	−1095.88648344
population (%)	23.28	20.75	15.51	10.28	6.9

**Table 2:** SET from urate(H_2_O)*_n_*^−^ clusters to CO_3_(H_2_O)*_n_*^•−^ clusters in water at pH = 7.4. Reaction Gibbs free energy Δ_r_*G* in kcal/mol, Gibbs free energy of activation Δ*G*^≠^ in kcal/mol, transition state theory rate constant *k*^TST^ in M^−1^ s^−1^, and rate constant including molar fractions $k_{Mf}^{SET}$ in M^−1^ s^−1^. Vertical and adiabatic detachment energy VDE and ADE in eV of urate(H_2_O)*_n_*^−^ clusters, and adiabatic and vertical electron affinity AEA and VEA in eV of CO_3_(H_2_O)*_n_*^•−^ clusters are also given.

	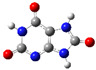 Urate^−^ VDE (ADE) = 5.49 (5.17)			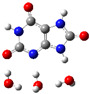 Urate(H_2_O)_3_^−^ 5.58 (5.25)			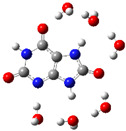 Urate(H_2_O)_6_^−^ 5.65 (5.27)			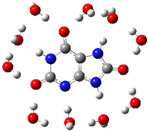 Urate(H_2_O)_10_^−^ 5.78 (5.41)		
CO_3_(H_2_O)*_n_*^•−^ *n*; AEA (VEA)	Δ_r_*G*	Δ*G*^≠^	*k* ^TST^ $k_{Mf}^{SET}$	Δ_r_*G*	Δ*G*^≠^	*k* ^TST^ $k_{Mf}^{SET}$	Δ_r_*G*	Δ*G*^≠^	*k* ^TST^ $k_{Mf}^{SET}$	Δ_r_*G*	Δ*G*^≠^	*k* ^TST^ $k_{Mf}^{SET}$
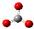 0; 5.38 (5.11)	−2.9	1.4	6.1 × 10^11^ 7.4 × 10^9^	−1.5	2.2	1.5 × 10^11^ 7.2 × 10^9^	−2.4	2.5	9.8 × 10^10^ 7.1 × 10^9^	2.5	4.6	2.7 × 10^9^ 2.0 × 10^9^
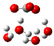 4; 5.74 (5.27)	−11.7	0.6	2.3 × 10^12^ 7.3 × 10^9^	−10.3	1.1	9.9 × 10^11^ 7.3 × 10^9^	−11.2	1.3	6.5 × 10^11^ 7.3 × 10^9^	−6.3	2.5	9.2 × 10^10^ 6.9 × 10^9^
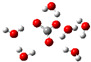 6; 6.01 (5.41)	−17.7	0.2	4.7 × 10^12^ 7.3 × 10^9^	−16.2	0.4	3.0 × 10^12^ 7.3 × 10^9^	−17.1	0.6	2.2 × 10^12^ 7.3 × 10^9^	−12.2	1.4	6.1 × 10^11^ 7.3 × 10^9^
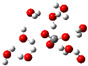 9; 6.21 (5.77)	−20.0	0.1	4.9 × 10^12^ 7.4 × 10^9^	−18.6	0.0	6.2 × 10^12^ 7.3 × 10^9^	−19.5	0.0	6.1 × 10^12^ 7.3 × 10^9^	−14.6	0.3	3.9 × 10^12^ 7.3 × 10^9^
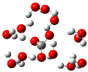 12; 6.28 (5.63)	−27.7	0.0	6.2 × 10^12^ 7.5 × 10^9^	−25.8	0.0	5.9 × 10^12^ 7.4 × 10^9^	−26.7	0.1	5.2 × 10^12^ 7.3 × 10^9^	−21.8	0.4	3.0 × 10^12^ 7.3 × 10^9^
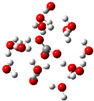 15; 6.50 (5.85)	−27.1	0.1	5.5 × 10^12^ 7.4 × 10^9^	−25.7	0.0	6.2 × 10^12^ 7.4 × 10^9^	−26.6	0.0	6.0 × 10^12^ 7.3 × 10^9^	−21.7	0.2	4.1 × 10^12^ 7.3 × 10^9^
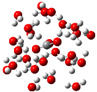 24; 6.62 (5.94)	−30.5	0.6	2.2 × 10^12^ 7.6 × 10^9^	−29.0	0.3	4.0 × 10^12^ 7.4 × 10^9^	−29.9	0.1	5.2 × 10^12^ 7.5 × 10^9^	−25.0	0.0	6.2 × 10^12^ 7.4 × 10^9^

**Table 3:** Calculated thermodynamic and binding parameters of the most stable urate conformers within the active sites of SOD1 and OGG1, including the following: inhibition constants (*K*_i_, µM) and binding free energy (Δ*G*_bind_, kcal mol^−1^) values obtained from various energy components such as total internal energy (Δ*G*_total_), torsional free energy (Δ*G*_tor_), unbound system’s energy (Δ*G*_unb_), electrostatic energy (Δ*G*_elec_), and the sum of dispersion and repulsion (Δ*G*_vdw_), hydrogen bond (Δ*G*_hbond_), and desolvation (Δ*G*_desolv_).

Complex	Δ*G_bind_*	*K*_i_ (mM)	Δ*G_inter_*	Δ*G_vdw+hbond+desolv_*	Δ*G_elec_*	Δ*G_total_*	Δ*G_tor_*	Δ*G_unb_*
SOD1-urate	−5.25	0.14	−5.25	−5.13	−0.12	0.00	0.00	0.00
SOD1-LSC-1	−3.62	0.22	−3.90	−3.93	0.03	−0.08	0.27	−0.08
OGG1-urate	−3.99	1.19	−3.99	−3.94	−0.05	0.00	0.00	0.00
OGG1-TH13264	−7.44	3.52	−8.04	−8.00	−0.04	−0.28	0.60	−0.28

## Data Availability

The original contributions presented in this study are included in the article/Supplementary Material. Further inquiries can be directed to the corresponding author.

## Supplementary Materials

The following supporting information can be downloaded at: https://www.mdpi.com/article/10.3390/antiox15060761/s1, Tables S1–S8: Optimized geometry and Cartesian coordinates of investigated clusters; Table S9: Spin-squared values 〈*S*^2^〉; Tables S10–S13: Complete results of modeled SET reactions; Figure S1 and Tables S14 and S15: additional data. Reference [72] is cited in the Supplementary Material.
