# Supplementary material for: Urate as a CO3•− Scavenger and Regulator of SOD-1 and OGG1 Enzymes: Insights from DFT, Molecular Docking, and Molecular Dynamics
Source: Antioxidants (Basel). 2026 Jun 16;15(6):761. doi: 10.3390/antiox15060761 (PMC13296194; doi:10.3390/antiox15060761)
Supplement: Supplementary file 1 [file antioxidants-15-00761-s001.zip › antioxidants-4322867-supplementary.pdf]

SUPPLEMENTARY MATERIAL 2026

Urate as a $CO_3^{\bullet-}$ scavenger and regulator of SOD-1 and OGG1 enzymes: insights from DFT, molecular docking, and molecular dynamics

Ana Amić[a,*], Žiko Milanović[b], Denisa Mastiľák Cagardová[c]

[a]*Department of Chemistry, Josip Juraj Strossmayer University of Osijek, Ulica cara Hadrijana 8A, 31000 Osijek, Croatia*
[b]*University of Kragujevac, Institute for Information Technologies, Department of Science, Liceja Kneževine Srbija 1A, 34000 Kragujevac, Serbia*
[c]*Institute of Physical Chemistry and Chemical Physics, Department of Chemical Physics, Slovak University of Technology in Bratislava, Radlinského 9, SK-812 37 Bratislava, Slovakia*

[*]Corresponding author.
E-mail address: aamic@kemija.unios.hr

Table of contents

1.	Table S1. The fully optimized minimal energy structures of urate$(H_2O)_3^-$ clusters calculated at SMD/M06-2X/6-311++G(d,p) level of theory. Solvation energy E^{solv} (kcal mol^{-1}), the total energy of the cluster E (*a.u.*), and Boltzmann population (%).	3
2.	Table S2. The fully optimized minimal energy structures of urate$(H_2O)_6^-$ clusters calculated at SMD/M06-2X/6-311++G(d,p) level of theory. Solvation energy E^{solv} (kcal mol^{-1}), the total energy of the cluster E (*a.u.*), and Boltzmann population (%).	4
3.	Table S3. Optimized geometry and Cartesian coordinates of: a) free urate; b) urate$(H_2O)_3^-$ at M06-2X/6-311++G(d,p) level of theory.	5
4.	Table S4. Optimized geometry and Cartesian coordinates of: a) urate$(H_2O)_6^-$; b) urate$(H_2O)_{10}^-$ at M06-2X/6-311++G(d,p) level of theory.	6
5.	Table S5. Optimized geometry and Cartesian coordinates of: a) free $CO_3^{\bullet-}$; b) $CO_3(H_2O)_4^{\bullet-}$ conformer at M06-2X/6-311++G(d,p) level of theory.	7
6.	Table S6. Optimized geometry and Cartesian coordinates of: a) $CO_3(H_2O)_6^{\bullet-}$ conformer; b) $CO_3(H_2O)_9^{\bullet-}$ conformer at M06-2X/6-311++G(d,p) level of theory.	8
7.	Table S7. Optimized geometry and Cartesian coordinates of: a) $CO_3(H_2O)_{12}^{\bullet-}$ conformer; b) $CO_3(H_2O)_{15}^{\bullet-}$ conformer at M06-2X/6-311++G(d,p) level of theory.	9
8.	Table S8. Optimized geometry and Cartesian coordinates of $CO_3(H_2O)_{24}^{\bullet-}$ conformer at M06-2X/6-311++G(d,p) level of theory.	10
9.	Table S9. Spin-squared values $\langle S^2 \rangle$ before and after annihilation of the first spin contaminant for studied radical species.	12

10.	Table S10. SET from unhydrated urate^{-} to $CO_3(H_2O)_n^{\bullet-}$ species in water at pH = 7.4. Reaction Gibbs free energy Δ_rG in kcal/mol, Gibbs free energy of activation $\Delta G^{\neq}$ in kcal/mol, reorganization energy λ in kcal/mol, transition state theory rate constant k^{TST} in M^{-1} s^{-1}, diffusion rate constant k_D in M^{-1} s^{-1}, apparent rate constant k_{app} in M^{-1} s^{-1}, and rate constant including molar fractions k_{Mf} in M^{-1} s^{-1}.	13
11.	Table S11. SET from urate$(H_2O)_3^{-}$ cluster to $CO_3(H_2O)_n^{\bullet-}$ species in water at pH = 7.4. Reaction Gibbs free energy Δ_rG in kcal/mol, Gibbs free energy of activation $\Delta G^{\neq}$ in kcal/mol, reorganization energy λ in kcal/mol, transition state theory rate constant k^{TST} in M^{-1} s^{-1}, diffusion rate constant k_D in M^{-1} s^{-1}, apparent rate constant k_{app} in M^{-1} s^{-1}, and rate constant including molar fractions k_{Mf} in M^{-1} s^{-1}.	14
12.	Table S12. SET from urate$(H_2O)_6^{-}$ cluster to $CO_3(H_2O)_n^{\bullet-}$ species in water at pH = 7.4. Reaction Gibbs free energy Δ_rG in kcal/mol, Gibbs free energy of activation $\Delta G^{\neq}$ in kcal/mol, reorganization energy λ in kcal/mol, transition state theory rate constant k^{TST} in M^{-1} s^{-1}, diffusion rate constant k_D in M^{-1} s^{-1}, apparent rate constant k_{app} in M^{-1} s^{-1}, and rate constant including molar fractions k_{Mf} in M^{-1} s^{-1}.	15
13.	Table S13. SET from urate$(H_2O)_{10}^{-}$ cluster to $CO_3(H_2O)_n^{\bullet-}$ species in water at pH = 7.4. Reaction Gibbs free energy Δ_rG in kcal/mol, Gibbs free energy of activation $\Delta G^{\neq}$ in kcal/mol, reorganization energy λ in kcal/mol, transition state theory rate constant k^{TST} in M^{-1} s^{-1}, diffusion rate constant k_D in M^{-1} s^{-1}, apparent rate constant k_{app} in M^{-1} s^{-1}, and rate constant including molar fractions k_{Mf} in M^{-1} s^{-1}.	16
14.	Figure S1. Plot of rate constants (log k^{TST}) vs reaction Gibbs free energy (Δ_rG) for SET from urate$(H_2O)_3^{-}$ cluster to $CO_3(H_2O)_n^{\bullet-}$ clusters (n = 0, 4, 6 and 9).	17
15.	Table S14. Electron affinity (EA in eV) of $CO_3(H_2O)n^{\bullet-}$ clusters *vs* log k^{TST} for reaction with urate$(H_2O)n^{-}$ clusters.	17
16.	Table S15. Vertical detachment energy (VDE in eV) of urate$(H_2O)n^{-}$ clusters *vs* log k^{TST} for reaction with $CO_3(H_2O)n^{\bullet-}$ clusters.	17

Table S1. The fully optimized minimal energy structures of urate$(H_2O)_3^-$ clusters calculated at SMD/M06-2X/6-311++G(d,p) level of theory. Solvation energy E^{solv} (kcal mol^{-1}), the total energy of the cluster E (*a.u.*), and Boltzmann population (%).

	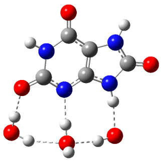	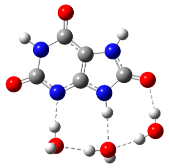	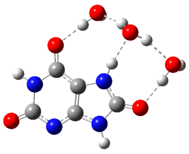	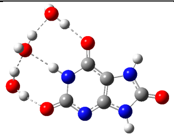	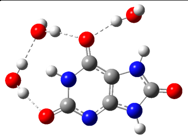
E^{solv} (kcal mol^{-1})	-17.55	-17.46	-17.39	-16.97	-16.82
E (*a.u.*)	-866.55542497	-866.55527264	-866.55515990	-866.55449004	-866.55426240
population (%)	26.52	22.53	19.97	9.75	7.64
	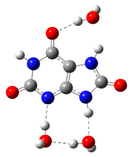	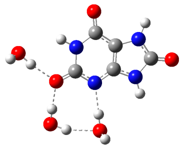	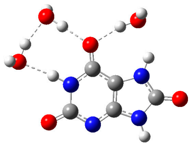	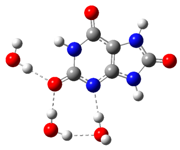	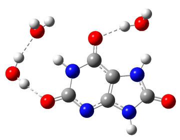
E^{solv} (kcal mol^{-1})	-16.63	-15.87	-15.85	-15.83	-15.74
E (*a.u.*)	-866.55395796	-866.55274624	-866.55271441	-866.55267090	-866.55254054
population (%)	5.52	1.51	1.46	1.39	1.21
	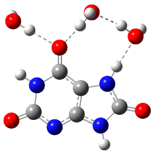	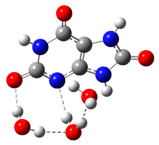	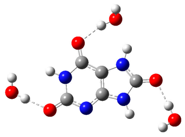	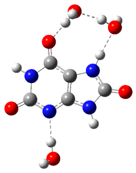	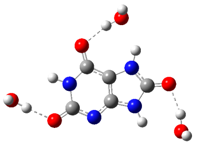
E^{solv} (kcal mol^{-1})	-15.35	-15.25	-15.12	-15.05	-14.95
E (*a.u.*)	-866.55191744	-866.55175614	-866.55154065	-866.55142776	-866.55127606
population (%)	0.62	0.52	0.41	0.37	0.31
	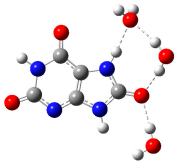	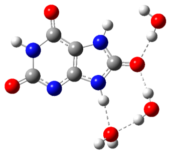	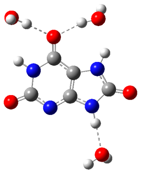	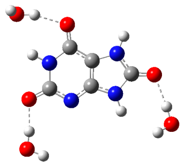	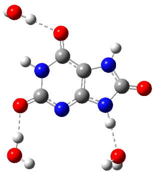
E^{solv} (kcal mol^{-1})	-14.37	-14.19	-13.65	-13.45	-12.72
E (*a.u.*)	-866.55035983	-866.55007120	-866.54920169	-866.54888061	-866.54772706
population (%)	0.12	0.09	0.03	0.02	0.01

Table S2. The fully optimized minimal energy structures of urate$(H_2O)_6^-$ clusters calculated at SMD/M06-2X/6-311++G(d,p) level of theory. Solvation energy E^{solv} (kcal mol^{-1}), the total energy of the cluster E (*a.u.*), and Boltzmann population (%).

	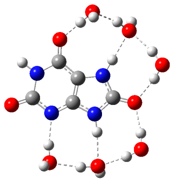	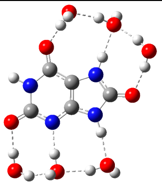	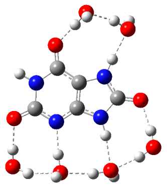	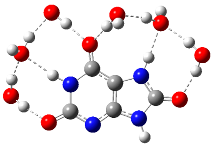	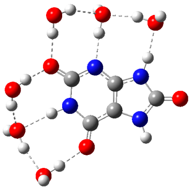
E^{solv} (kcal mol^{-1})	-35.10	-35.04	-34.86	-34.62	-34.39
E (*a.u.*)	-1095.88761877	-1095.88751127	-1095.88723963	-1095.88685498	-1095.88648344
population (%)	23.28	20.75	15.51	10.28	6.9
	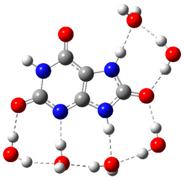	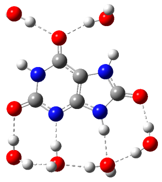	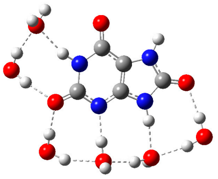	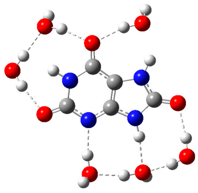	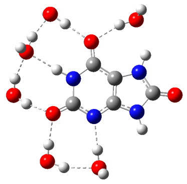
E^{solv} (kcal mol^{-1})	-34.37	-34.23	-33.89	-33.84	-33.70
E (*a.u.*)	-1095.88644992	-1095.88623498	-1095.88568312	-1095.88559913	-1095.88539072
population (%)	6.66	5.29	2.93	2.68	2.14
	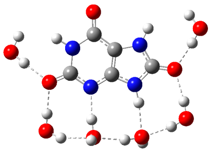	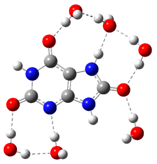	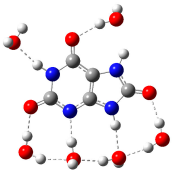	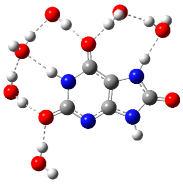	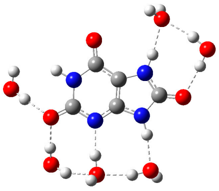
E^{solv} (kcal mol^{-1})	-33.17	-33.05	-33.02	-32.64	-32.44
E (*a.u.*)	-1095.88453687	-1095.88435501	-1095.8842942	-1095.88369578	-1095.88338130
population (%)	0.86	0.71	0.66	0.35	0.25
	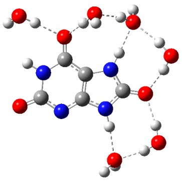	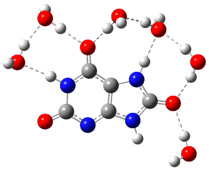	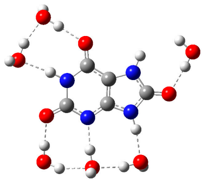	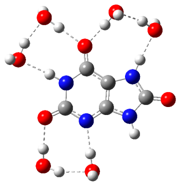	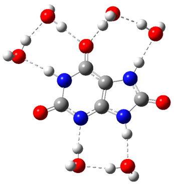
E^{solv} (kcal mol^{-1})	-32.11	-32.07	-32.05	-31.99	-31.69
E (*a.u.*)	-1095.88285314	-1095.88278854	-1095.88274708	-1095.88265072	-1095.88218435
population (%)	0.14	0.13	0.13	0.11	0.07
	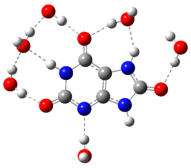	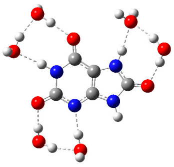	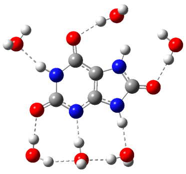	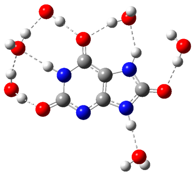	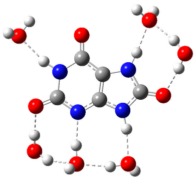
E^{solv} (kcal mol^{-1})	-31.59	-31.24	-31.13	-30.98	-30.62
E (*a.u.*)	-1095.88202865	-1095.88147045	-1095.88128343	-1095.88105303	-1095.88046815
population (%)	0.06	0.03	0.03	0.02	0.01

	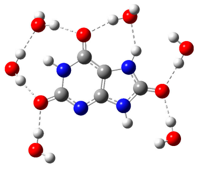	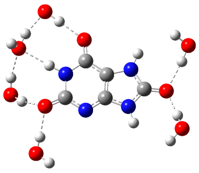	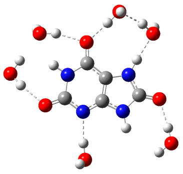	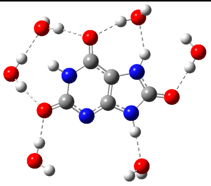	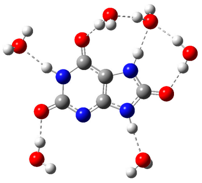
E^{solv} (kcal mol^{-1})	-30.26	-30.26	-29.91	-29.64	-28.83
E (*a.u.*)	-1095.87990697	-1095.87990189	-1095.87934526	-1095.87891751	-1095.87762918
population (%)	0.01	0.01	0.00	0.00	0.00

Table S3. Optimized geometry and Cartesian coordinates of: a) free urate^{-}; b) urate$(H_2O)_3^{-}$ at M06-2X/6-311++G(d,p) level of theory.

a) free urate^{-}

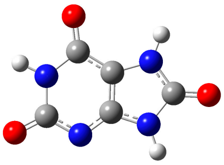

6	-0.321906000	0.596201000	-0.000075000
6	-0.214501000	-0.780277000	-0.000104000
6	2.038849000	-0.814815000	-0.000444000
6	0.842249000	1.381852000	0.000181000
6	-2.419882000	-0.229855000	-0.000265000
7	1.979065000	0.580655000	0.000083000
7	0.893014000	-1.524530000	-0.000039000
7	-1.685101000	0.914484000	-0.000150000
7	-1.503687000	-1.268016000	-0.000199000
1	2.870452000	1.064462000	0.000448000
1	-2.091688000	1.839547000	-0.000168000
1	-1.774895000	-2.242892000	-0.000216000
8	0.933769000	2.622916000	0.000397000
8	3.171837000	-1.342410000	0.000775000
8	-3.647576000	-0.342746000	-0.000382000

b) urate$(H_2O)_3^{-}$

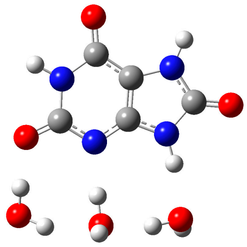

6	-1.608523000	-0.662427000	-0.013082000
6	-0.481866000	0.131147000	0.003933000
6	0.974987000	-1.602432000	0.085018000
6	-1.479211000	-2.062209000	0.022340000
6	-2.291365000	1.484423000	-0.072884000
7	-0.137943000	-2.436065000	0.068217000
7	0.797973000	-0.267736000	0.049586000
7	-0.908675000	1.436689000	-0.032437000
7	-2.715426000	0.190161000	-0.061352000
8	-2.379662000	-2.917593000	0.016141000
8	4.266567000	-0.581152000	-0.649203000
1	4.003759000	0.290439000	-0.319096000
1	2.112089000	0.900680000	0.442291000
1	3.024178000	1.577606000	1.507947000
8	2.805264000	1.594618000	0.569347000
1	1.183851000	3.956235000	-0.929933000
1	1.736003000	3.001109000	0.147519000
1	0.054002000	-3.432075000	0.093170000
8	0.992515000	3.601809000	-0.054995000
1	-0.306896000	2.268113000	-0.052229000
8	-2.984993000	2.503560000	-0.107577000
8	2.103797000	-2.141671000	0.132517000
1	3.518333000	-1.150217000	-0.383186000
1	-3.688851000	-0.080807000	-0.076379000

Table S4. Optimized geometry and Cartesian coordinates of: a) urate$(H_2O)_6^-$; b) urate$(H_2O)_{10}^-$ at M06-2X/6-311++G(d,p) level of theory.

a) urate$(H_2O)_6^-$

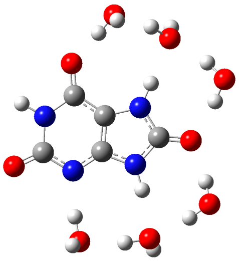

6	-0.454161000	-0.811674000	-0.235766000
6	0.889677000	-0.492140000	-0.205128000
6	1.611513000	-2.633132000	-0.020342000
6	-0.838450000	-2.158728000	-0.149449000
6	-0.292030000	1.418903000	-0.383302000
7	0.264934000	-2.996325000	-0.054574000
7	1.930907000	-1.323684000	-0.106647000
7	0.973130000	0.875878000	-0.297739000
7	-1.169360000	0.387621000	-0.338302000
8	-1.990629000	-2.639164000	-0.153342000
1	3.600580000	-0.698231000	0.248463000
1	-4.265605000	-0.136394000	-0.023771000
1	4.676976000	-0.394718000	1.324579000
8	-4.052354000	0.658942000	-0.555871000
8	4.457563000	-0.235301000	0.400327000
1	-3.423401000	-1.964091000	0.651523000
1	-4.108080000	-1.378337000	1.906917000
8	-4.264443000	-1.588619000	0.979913000
1	3.887973000	2.507705000	-1.074001000
1	3.970256000	1.434432000	0.042175000
1	0.068480000	-3.989504000	0.012219000
8	3.533486000	2.275956000	-0.208531000
1	1.835486000	1.430357000	-0.310241000
8	-0.550149000	2.631367000	-0.481012000
8	2.462714000	-3.535289000	0.089422000
1	-2.182757000	0.497814000	-0.443289000
1	-4.480085000	0.537553000	-1.410439000
8	1.563344000	4.115619000	0.757262000
8	-3.195628000	3.183262000	0.371176000
1	2.301132000	3.536828000	0.511098000
1	-3.606036000	2.347843000	0.095847000
1	0.790645000	3.701823000	0.339035000
1	-2.273379000	3.088925000	0.079879000

b) urate$(H_2O)_{10}^-$

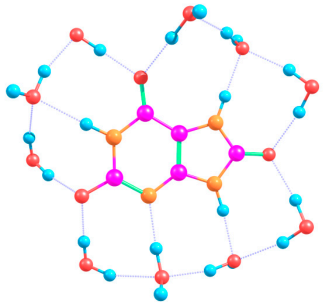

6	-0.298145000	-0.601990000	-0.162537000
6	-0.480317000	0.764066000	-0.071290000
6	1.702180000	1.224034000	0.296477000
6	0.991260000	-1.129182000	-0.013131000
6	-2.483450000	-0.195036000	-0.439328000
7	1.936647000	-0.141394000	0.206275000
7	0.451763000	1.696520000	0.150790000
7	-1.820019000	0.999755000	-0.244676000
7	-1.551719000	-1.178236000	-0.390494000
8	1.328739000	-2.335267000	-0.059457000
8	2.329859000	4.608260000	-0.368474000
8	4.935953000	1.198977000	-1.004705000
1	1.412132000	4.776197000	-0.109942000
1	5.031929000	0.356176000	-0.533194000
1	-0.066462000	3.402431000	0.490936000
1	-1.412354000	-4.318907000	-0.054865000
1	-0.387140000	4.515190000	1.528222000
8	-2.140874000	-4.008812000	-0.632488000
8	-0.403775000	4.325538000	0.583073000
1	0.460068000	-3.679420000	0.727597000
1	2.911797000	-3.108421000	-0.304640000
1	-0.253363000	-4.302085000	1.947767000
1	3.991767000	-3.692362000	-1.243214000
8	3.801717000	-3.512218000	-0.316301000
8	-0.020671000	-4.474741000	1.029024000
1	-3.123897000	4.038901000	-1.128822000
1	-2.096703000	4.054772000	0.034050000
1	2.905675000	-0.447730000	0.350449000
8	-2.957742000	3.684191000	-0.248382000
1	-2.278460000	1.917349000	-0.262231000
8	4.718860000	-1.094233000	0.613815000
1	5.059379000	-1.150507000	1.513120000
1	4.555584000	-2.013609000	0.324033000
8	-3.706830000	-0.317245000	-0.616305000
8	2.685909000	1.973948000	0.514108000
1	2.482537000	3.693437000	-0.066503000
1	-1.762206000	-2.172575000	-0.523936000
1	-2.041291000	-4.471989000	-1.471131000
1	4.173813000	1.601292000	-0.554848000
8	-5.019237000	1.902796000	0.623491000
8	-4.563131000	-2.872569000	0.264498000

				1	-4.377169000	2.596943000	0.410381000
				1	-3.777615000	-3.373143000	-0.009596000
				1	-4.655446000	1.109154000	0.198131000
				1	-4.368651000	-1.967966000	-0.031355000

Table S5. Optimized geometry and Cartesian coordinates of: a) free $CO_3^{\bullet-}$; b) $CO_3(H_2O)_4^{\bullet-}$ conformer at M06-2X/6-311++G(d,p) level of theory.

a) free $CO_3^{\bullet-}$				b) $CO_3(H_2O)_4^{\bullet-}$			
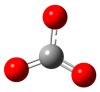				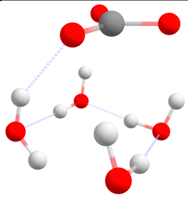			
8	1.071760000	-0.689263000	-0.000010000	8	-1.481537000	-1.236090000	-1.304268000
6	-0.011133000	-0.070204000	0.000036000	8	-1.384305000	-1.273872000	1.514308000
8	0.112359000	1.250233000	-0.000008000	8	1.528156000	-1.147404000	1.522865000
8	-1.175770000	-0.508317000	-0.000010000	8	1.339578000	-1.259662000	-1.297754000
				8	-0.132255000	1.269230000	1.150315000
				6	0.024621000	1.501015000	-0.054664000
				8	1.153015000	1.642701000	-0.654454000
				8	-0.901195000	1.638072000	-0.932085000
				1	-1.528946000	-0.277639000	-1.427686000
				1	-1.552811000	-1.346922000	-0.337534000
				1	-1.225911000	-0.315586000	1.506344000
				1	-0.486458000	-1.624342000	1.620394000
				1	1.181993000	-0.248617000	1.626217000
				1	1.537283000	-1.272865000	0.555964000
				1	1.525878000	-0.316505000	-1.400369000
				1	0.367894000	-1.318415000	-1.370691000

Table S6. Optimized geometry and Cartesian coordinates of: a) $CO_3(H_2O)_6^{\bullet-}$ conformer; b) $CO_3(H_2O)_9^{\bullet-}$ conformer at M06-2X/6-311++G(d,p) level of theory.

a) $CO_3(H_2O)_6^{\bullet-}$

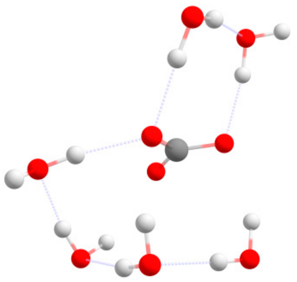

6	-0.397155000	0.148242000	-0.353021000
8	-0.311595000	-0.744493000	0.503775000
8	0.341614000	0.233932000	-1.406194000
8	-1.201549000	1.138564000	-0.356674000
8	3.286368000	-0.355825000	-0.992436000
1	2.683579000	0.126161000	-1.571152000
1	2.814976000	-1.189740000	-0.799800000
8	-3.902874000	0.280258000	-0.835667000
1	-3.039577000	0.720403000	-0.795636000
8	0.985635000	2.988205000	0.608630000
1	0.159416000	2.678964000	0.216362000
1	1.412234000	2.174015000	0.932169000
8	1.755285000	-2.533699000	-0.157473000
1	0.998468000	-1.990624000	0.130644000
1	2.125004000	-2.901162000	0.652949000
8	-3.006006000	-1.512091000	1.195716000
1	-2.065913000	-1.301580000	1.087707000
8	2.198006000	0.568884000	1.462863000
1	2.673652000	0.313846000	0.650968000
1	1.441503000	-0.033529000	1.485595000
1	-4.539359000	0.957323000	-0.581888000
1	-3.440116000	-0.953410000	0.529894000

b) $CO_3(H_2O)_9^{\bullet-}$

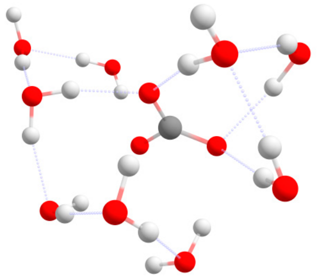

8	-1.213516000	-0.478506000	-1.421863000
8	0.814112000	0.246856000	-1.365320000
8	0.046986000	-0.737334000	0.437589000
8	2.370229000	0.522270000	1.498220000
1	1.609077000	-0.010130000	1.209697000
1	2.220993000	1.377175000	1.057826000
8	1.360663000	2.803784000	0.120964000
1	1.241435000	2.329875000	-0.712167000
1	0.529615000	2.633714000	0.602659000
8	3.127899000	-1.725363000	-1.558140000
1	3.669574000	-1.448423000	-0.797201000
1	2.419787000	-1.071925000	-1.602351000
8	-3.604287000	0.174460000	0.159563000
1	-2.915871000	0.014061000	-0.501950000
1	-3.276319000	-0.307225000	0.935315000
8	-1.106348000	2.048760000	1.289957000
1	-1.004442000	1.101003000	1.445986000
1	-1.493534000	2.112665000	0.396445000
8	-2.166753000	-1.447323000	1.993609000
1	-1.992963000	-1.616031000	2.925801000
1	-1.307512000	-1.226842000	1.590611000
8	-1.727297000	2.395581000	-1.435788000
1	-0.916209000	2.844727000	-1.701907000
1	-1.620780000	1.489151000	-1.760723000
8	4.611802000	-0.815582000	0.655051000
1	3.854214000	-0.319012000	1.027792000
1	4.827809000	-1.483523000	1.314124000
8	-2.524230000	-2.913630000	-0.453004000
1	-2.548427000	-2.623474000	0.471298000
1	-2.117601000	-2.161496000	-0.908297000
6	-0.182155000	-0.371011000	-0.726612000

Table S7. Optimized geometry and Cartesian coordinates of: a) $CO_3(H_2O)_{12}^{\bullet -}$ conformer; b) $CO_3(H_2O)_{15}^{\bullet -}$ conformer at M06-2X/6-311++G(d,p) level of theory.

a) $CO_3(H_2O)_{12}^{\bullet -}$				b) $CO_3(H_2O)_{15}^{\bullet -}$			
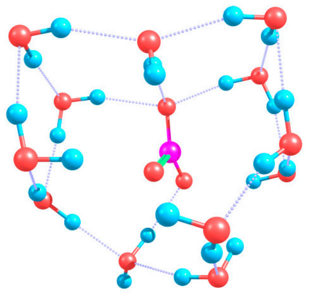				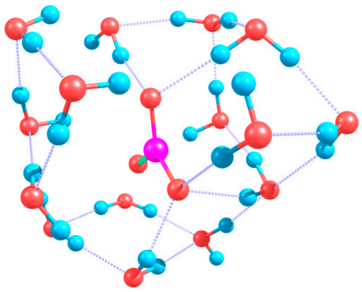			
8	0.325302000	0.574082000	0.153820000	8	0.973873000	-0.397717000	0.312220000
8	-0.143288000	-0.891850000	-1.377530000	8	-0.715179000	0.930642000	0.121498000
8	-0.591990000	1.299283000	-1.687011000	8	-0.192632000	-0.498055000	-1.544427000
8	-2.851766000	-1.630581000	-1.880331000	8	-0.629243000	2.986498000	-1.926435000
1	-1.897356000	-1.461408000	-1.933634000	1	-1.123696000	2.302336000	-1.454210000
1	-3.214233000	-0.764664000	-1.624870000	1	0.234352000	2.562552000	-2.093945000
8	-0.715278000	1.103750000	3.039799000	8	4.026487000	-0.115146000	-0.173978000
1	0.183623000	0.758552000	3.019404000	1	3.590834000	0.718230000	0.050307000
1	-0.724606000	1.830074000	2.385719000	1	3.434448000	-0.554401000	-0.814086000
8	3.133449000	-2.683627000	0.451130000	8	-2.431787000	0.179175000	3.106409000
1	2.196423000	-2.806171000	0.680192000	1	-1.816959000	0.931638000	3.103102000
1	3.097527000	-2.221454000	-0.407486000	1	-2.932313000	0.273984000	2.274429000
8	-2.638988000	-0.172697000	1.429329000	8	2.395574000	2.204641000	0.753472000
1	-2.527492000	-1.125480000	1.263304000	1	1.768130000	2.953561000	0.683126000
1	-1.895003000	0.111285000	1.989509000	1	1.918548000	1.542996000	1.270240000
8	3.257890000	1.373826000	-0.651071000	8	-3.860422000	-1.850614000	-1.154798000
1	3.165215000	0.938470000	0.216694000	1	-3.094647000	-2.387144000	-0.893206000
1	2.548757000	2.039332000	-0.679028000	1	-3.504783000	-1.236874000	-1.822557000
8	2.957858000	-0.154912000	1.701384000	8	-0.893820000	-1.923680000	2.115796000
1	3.131092000	-1.051903000	1.358427000	1	-1.392722000	-1.169063000	2.489055000
1	2.001843000	-0.132288000	1.825784000	1	-0.004812000	-1.585056000	1.950872000
8	-1.080735000	3.043404000	1.071197000	8	2.425064000	-1.744538000	-1.831345000
1	-1.624912000	2.535532000	0.455298000	1	1.564906000	-1.328031000	-1.995543000
1	-0.268995000	3.227500000	0.568328000	1	2.208331000	-2.441707000	-1.185147000
8	2.728911000	-1.118910000	-1.851496000	8	-3.488675000	0.424069000	0.523992000
1	2.892711000	-0.211240000	-1.530714000	1	-3.710858000	-0.398843000	0.053093000
1	1.763920000	-1.202769000	-1.849805000	1	-2.592199000	0.640219000	0.219067000
8	-2.498423000	-2.890524000	0.642456000	8	0.381730000	4.120374000	0.499488000
1	-2.653262000	-2.577259000	-0.267566000	1	-0.030601000	3.800482000	-0.324144000
1	-1.532761000	-2.943605000	0.729692000	1	-0.122488000	3.703582000	1.214900000
8	0.329874000	-2.446350000	0.927823000	8	-0.471539000	2.202111000	2.562110000
1	0.241214000	-1.965865000	0.083050000	1	-0.383656000	1.672357000	1.747717000
1	0.260352000	-1.754573000	1.599280000	1	0.374940000	2.118096000	3.016868000
8	-3.386837000	1.042476000	-0.929372000	8	1.928286000	1.850001000	-2.041262000
1	-3.093712000	0.697416000	-0.061825000	1	2.130303000	1.883065000	-1.089396000
1	-2.568064000	1.334662000	-1.355440000	1	2.037624000	0.924670000	-2.290046000
8	1.139632000	3.296186000	-0.744460000	8	-2.686816000	0.262839000	-2.638545000
1	0.537663000	2.776322000	-1.304993000	1	-1.784296000	0.089994000	-2.315101000
1	1.293656000	4.123316000	-1.215014000	1	-3.010216000	0.981412000	-2.081742000
6	-0.173085000	0.316296000	-1.051608000	6	-0.042051000	0.036711000	-0.430155000
				8	1.451940000	-3.582616000	0.085512000

	1	0.495262000	-3.498225000	-0.079941000
	1	1.643983000	-4.526167000	0.039750000
	8	-1.282783000	-2.955756000	-0.414196000
	1	-1.287256000	-2.546523000	0.476664000
	1	-0.964797000	-2.252990000	-1.002790000
	8	3.147248000	-2.032695000	1.772506000
	1	2.511666000	-2.530958000	1.233758000
	1	3.476827000	-1.339721000	1.175715000

Table S8. Optimized geometry and Cartesian coordinates of $CO_3(H_2O)_{24}^{\bullet -}$ conformer at M06-2X/6-311++G(d,p) level of theory.

$CO_3(H_2O)_{24}^{\bullet -}$

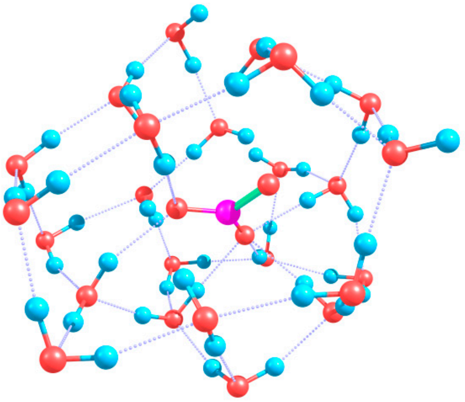

8	-0.793659000	1.274204000	-0.486966000
8	1.009072000	0.161359000	-0.013732000
8	-0.979415000	-0.884285000	-0.233300000
8	-2.357009000	-0.921780000	-2.755893000
1	-2.082771000	-0.039325000	-3.083913000
1	-1.993023000	-0.955178000	-1.856520000
8	0.146932000	-2.023826000	-3.568559000
1	-0.774288000	-1.747215000	-3.417621000
1	0.361612000	-2.566647000	-2.790531000
8	1.983666000	1.477494000	2.393116000
1	1.432413000	1.636697000	3.182171000
1	1.427338000	0.961755000	1.789793000
8	-4.623308000	-2.004576000	-1.386978000
1	-4.071935000	-2.571334000	-0.821983000
1	-4.002119000	-1.654445000	-2.046750000
8	2.199365000	2.802519000	-0.149207000
1	1.746477000	1.966018000	-0.343968000
1	2.278428000	2.765378000	0.818075000
8	3.055023000	-1.734416000	-0.817091000
1	3.523305000	-1.412828000	-1.609899000
1	2.574572000	-0.959797000	-0.484488000
8	0.694278000	-3.167478000	-1.005210000
1	0.118310000	-2.455359000	-0.681132000

1	1.589919000	-2.777691000	-0.968035000
8	0.092025000	1.912877000	4.445130000
1	-0.435707000	1.209136000	4.022049000
1	-0.258013000	2.725626000	4.057393000
8	-0.959046000	3.147070000	1.955283000
1	-0.615003000	3.688160000	1.213689000
1	-0.502761000	2.300744000	1.859225000
8	-2.132837000	3.519450000	-1.915336000
1	-2.681022000	4.191101000	-2.336540000
1	-2.630656000	3.229133000	-1.118905000
8	-0.045409000	4.503868000	-0.287084000
1	0.758547000	3.964418000	-0.391886000
1	-0.677994000	4.177587000	-0.952041000
8	-1.236730000	1.463847000	-3.598734000
1	-1.516214000	2.179726000	-2.998580000
1	-0.319950000	1.251765000	-3.351911000
8	-2.684859000	-3.164676000	0.473198000
1	-2.087667000	-2.492152000	0.114461000
1	-3.031263000	-2.753110000	1.289443000
8	1.376964000	0.395648000	-3.039617000
1	1.535441000	0.417377000	-2.086094000
1	0.994091000	-0.492366000	-3.209442000
8	4.199355000	-0.281547000	-2.950447000
1	3.310346000	-0.015895000	-3.234787000
1	4.403971000	0.330073000	-2.215954000
8	-3.724515000	-0.001868000	0.404005000
1	-2.811819000	-0.274015000	0.213865000
1	-4.254567000	-0.549973000	-0.203678000
8	-0.207619000	-4.253679000	1.442588000
1	-1.133836000	-4.043168000	1.234012000
1	0.235086000	-4.168102000	0.581006000
8	0.903670000	-1.788321000	2.179512000
1	0.469848000	-2.636557000	1.951974000
1	1.097277000	-1.360074000	1.331054000
8	-1.188825000	-0.096759000	2.966485000
1	-1.590846000	0.274994000	2.170459000
1	-0.472079000	-0.687320000	2.655560000
8	4.298995000	0.004352000	1.794598000
1	3.512859000	0.460361000	2.142316000
1	4.093614000	-0.944546000	1.844306000
8	-3.603804000	-1.676711000	2.683713000
1	-2.772838000	-1.325848000	3.040749000
1	-3.846566000	-1.014698000	2.012354000
8	-3.344776000	2.774910000	0.432710000
1	-2.584694000	2.848572000	1.037870000
1	-3.551889000	1.823303000	0.399926000
8	4.581536000	1.280702000	-0.674584000
1	3.856478000	1.922040000	-0.597035000
1	4.520581000	0.757795000	0.148518000
8	3.589106000	-2.790591000	1.738383000
1	2.704807000	-2.600040000	2.089392000
1	3.493658000	-2.633842000	0.783709000
6	-0.220810000	0.104576000	-0.231590000

Table S9. Spin-squared values $\langle S^2 \rangle$ before and after annihilation of the first spin contaminant for studied radical species.

radical species		$\langle S^2 \rangle$ before/after
urate$^{\bullet}$		0.7620/0.7501
urate$(H_2O)_3^{\bullet}$		0.7611/0.7501
urate$(H_2O)_6^{\bullet}$		0.7611/0.7501
urate$(H_2O)_{10}^{\bullet}$		0.7605/0.7501
$CO_3^{\bullet-}$		0.7583/0.7500
$CO_3(H_2O)_4^{\bullet-}$		0.7605/0.7501
$CO_3(H_2O)_6^{\bullet-}$		0.7600/0.7500
$CO_3(H_2O)_9^{\bullet-}$		0.7567/0.7500
$CO_3(H_2O)_{12}^{\bullet-}$		0.7566/0.7500
$CO_3(H_2O)_{15}^{\bullet-}$		0.7566/0.7500
$CO_3(H_2O)_{24}^{\bullet-}$		0.7562/0.7500

In all cases, the deviations from the ideal value ($\langle S^2 \rangle = 0.75$ for a pure doublet) were lower than 1.60% and 0.01% before and after annihilation of the first spin contaminant. Because spin contamination can be considered negligible if the value of $\langle S^2 \rangle$ differs from 0.75 by less than 10% (Young, 2001), the obtained energy values of radical species studied in this work are reliable.

Young, D.C., *Computational Chemistry: A Practical Guide for Applying Techniques to Real-World Problems*, John Wiley & Sons, New York, 2001, p. 228. doi: 10.1002/0471220655.

Table S10. SET from unhydrated urate$^-$ to $CO_3(H_2O)_n^{\bullet -}$ species in water at pH = 7.4. Reaction Gibbs free energy Δ_rG in kcal/mol, Gibbs free energy of activation $\Delta G^{\neq}$ in kcal/mol, reorganization energy λ in kcal/mol, transition state theory rate constant k^{TST} in M^{-1} s^{-1}, diffusion rate constant k_D in M^{-1} s^{-1}, apparent rate constant k_{app} in M^{-1} s^{-1}, and rate constant including molar fractions k_{Mf} in M^{-1} s^{-1}.

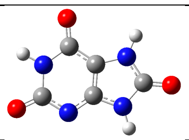							
	Δ_rG	$\Delta G^{\neq}$	λ	k^{TST}	k_D	k_{app}	k_{Mf}^{SET}
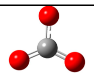	-2.9	1.4	10.5	6.1×10^{11}	7.6×10^{9}	7.5×10^{9}	7.40×10^{9}
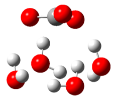	-11.7	0.6	18.4	2.3×10^{12}	7.4×10^{9}	7.4×10^{9}	7.30×10^{9}
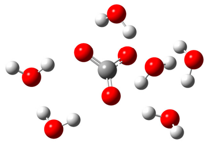	-17.7	0.2	21.3	4.7×10^{12}	7.4×10^{9}	7.4×10^{9}	7.30×10^{9}
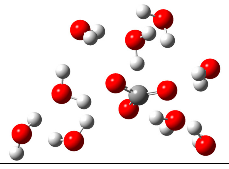	-20.0	0.1	16.9	4.9×10^{12}	7.5×10^{9}	7.5×10^{9}	7.40×10^{9}
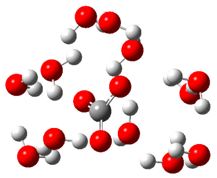	-27.7	0.0	26.6	6.2×10^{12}	7.6×10^{9}	7.6×10^{9}	7.50×10^{9}
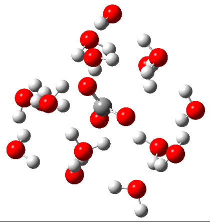	-27.1	0.1	24.6	5.5×10^{12}	7.5×10^{9}	7.5×10^{9}	7.40×10^{9}
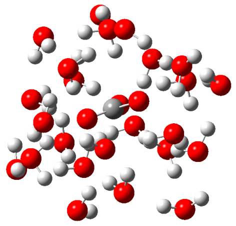	-30.5	0.6	22.9	2.2×10^{12}	7.8×10^{9}	7.7×10^{9}	7.59×10^{9}

Table S11. SET from urate$(H_2O)_3^-$ cluster to $CO_3(H_2O)_n^{\bullet -}$ species in water at pH = 7.4. Reaction Gibbs free energy $\Delta_r G$ in kcal/mol, Gibbs free energy of activation $\Delta G^{\neq}$ in kcal/mol, reorganization energy λ in kcal/mol, transition state theory rate constant k^{TST} in M^{-1} s^{-1}, diffusion rate constant k_D in M^{-1} s^{-1}, apparent rate constant k_{app} in M^{-1} s^{-1}, and rate constant including molar fractions k_{Mf} in M^{-1} s^{-1}.

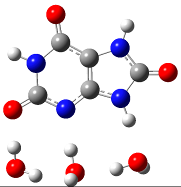							
	$\Delta_r G$	$\Delta G^{\neq}$	λ	k^{TST}	k_D	k_{app}	k_{Mf}^{SET}
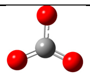	-1.5	2.2	11.7	1.5×10^{11}	7.7×10^9	7.3×10^9	7.20×10^9
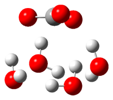	-10.3	1.1	19.5	9.9×10^{11}	7.4×10^9	7.4×10^9	7.30×10^9
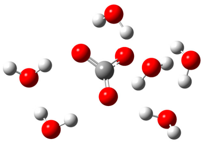	-16.2	0.4	22.5	3.0×10^{12}	7.4×10^9	7.4×10^9	7.30×10^9
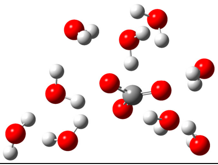	-18.6	0.0	18.0	6.2×10^{12}	7.4×10^9	7.4×10^9	7.30×10^9
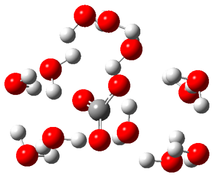	-25.8	0.0	27.7	5.9×10^{12}	7.5×10^9	7.5×10^9	7.40×10^9
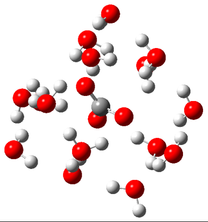	-25.7	0.0	25.7	6.2×10^{12}	7.5×10^9	7.5×10^9	7.40×10^9
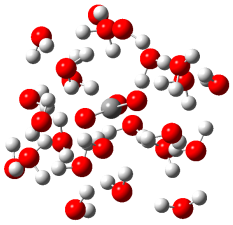	-29.0	0.3	24.0	4.0×10^{12}	7.6×10^9	7.6×10^9	7.50×10^9

Table S12. SET from urate$(H_2O)_6^-$ cluster to $CO_3(H_2O)_n^{\bullet -}$ species in water at pH = 7.4. Reaction Gibbs free energy Δ_rG in kcal/mol, Gibbs free energy of activation $\Delta G^{\neq}$ in kcal/mol, reorganization energy λ in kcal/mol, transition state theory rate constant k^{TST} in M^{-1} s^{-1}, diffusion rate constant k_D in M^{-1} s^{-1}, apparent rate constant k_{app} in M^{-1} s^{-1}, and rate constant including molar fractions k_{Mf} in M^{-1} s^{-1}.

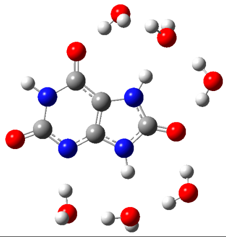

	Δ_rG	$\Delta G^{\neq}$	λ	k^{TST}	k_D	k_{app}	k_{Mf}^{SET}
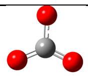	-2.4	2.5	14.2	9.8×10^{10}	7.7×10^9	7.2×10^9	7.10×10^9
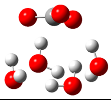	-11.2	1.3	22.1	6.5×10^{11}	7.5×10^9	7.4×10^9	7.30×10^9
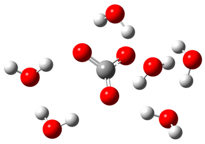	-17.1	0.6	25.0	2.2×10^{12}	7.4×10^9	7.4×10^9	7.30×10^9
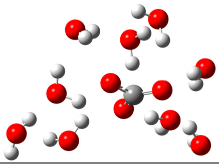	-19.5	0.0	20.6	6.1×10^{12}	7.4×10^9	7.4×10^9	7.30×10^9
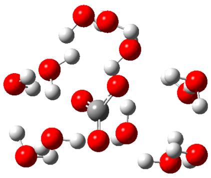	-26.7	0.1	30.3	5.2×10^{12}	7.4×10^9	7.4×10^9	7.30×10^9
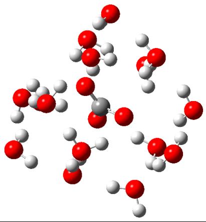	-26.6	0.0	28.3	6.0×10^{12}	7.4×10^9	7.4×10^9	7.30×10^9
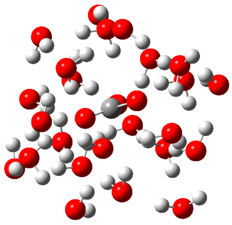	-29.9	0.1	26.6	5.2×10^{12}	7.6×10^9	7.6×10^9	7.50×10^9

Table S13. SET from urate$(H_2O)_{10}^-$ cluster to $CO_3(H_2O)_n^{\bullet-}$ species in water at pH = 7.4. Reaction Gibbs free energy Δ_rG in kcal/mol, Gibbs free energy of activation $\Delta G^{\neq}$ in kcal/mol, reorganization energy λ in kcal/mol, transition state theory rate constant k^{TST} in M^{-1} s^{-1}, diffusion rate constant k_D in M^{-1} s^{-1}, apparent rate constant k_{app} in M^{-1} s^{-1}, and rate constant including molar fractions k_{Mf} in M^{-1} s^{-1}.

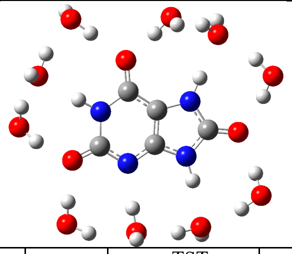

	Δ_rG	$\Delta G^{\neq}$	λ	k^{TST}	k_D	k_{app}	k_{Mf}^{SET}
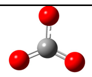	2.5	4.6	12.8	2.7×10^9	7.9×10^9	2.0×10^9	1.97×10^9
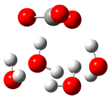	-6.3	2.5	20.6	9.2×10^{10}	7.5×10^9	7.0×10^9	6.90×10^9
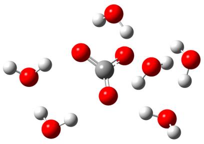	-12.2	1.4	23.6	6.1×10^{11}	7.5×10^9	7.4×10^9	7.30×10^9
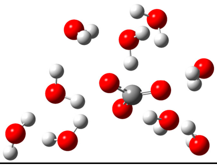	-14.6	0.3	19.2	3.9×10^{12}	7.5×10^9	7.4×10^9	7.3×10^9
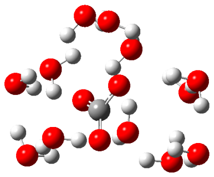	-21.8	0.4	28.8	3.0×10^{12}	7.4×10^9	7.4×10^9	7.3×10^9
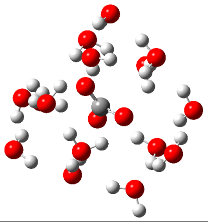	-21.7	0.2	26.8	4.1×10^{12}	7.4×10^9	7.4×10^9	7.3×10^9
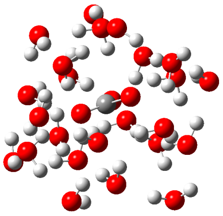	-25.0	0.0	25.1	6.2×10^{12}	7.5×10^9	7.5×10^9	7.4×10^9

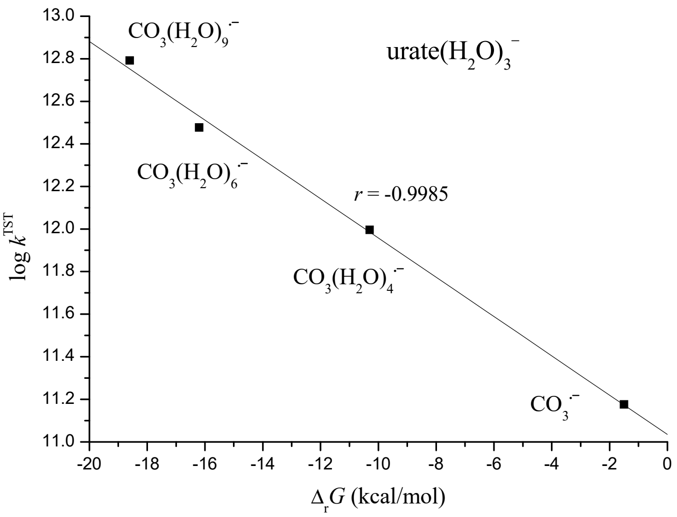

Figure S1. Plot of rate constants (log k^{TST}) vs reaction Gibbs free energy ($\Delta_r G$) for SET from urate$(H_2O)_3^-$ cluster to $CO_3(H_2O)_n^{•-}$ clusters (n = 0, 4, 6 and 9).

Table S14. Electron affinity (EA in eV) of $CO_3(H_2O)n^{•-}$ clusters *vs* log k^{TST} for reaction with urate$(H_2O)n^-$ clusters.

$CO_3(H_2O)n^{•-}$			log k^{TST}			
n	EA		urate⁻	urate$(H_2O)_3^-$	urate$(H_2O)_6^-$	urate$(H_2O)_{10}^-$
0	5.38		11.785	11.176	10.991	9.431
4	5.74		12.362	11.996	11.813	10.964
6	6.01		12.672	12.477	12.342	11.785
9	6.21		12.690	12.792	12.785	12.591
12	6.28		12.792	12.771	12.716	12.477
15	6.50		12.740	12.792	12.778	12.613
24	6.62		12.342	12.602	12.716	12.792

Table S15. Vertical detachment energy (VDE in eV) of urate$(H_2O)n^-$ clusters *vs* log k^{TST} for reaction with $CO_3(H_2O)n^{•-}$ clusters.

urate$(H_2O)n^-$			$CO_3(H_2O)n^{•-}$						
			log k^{TST}						
n	VDE		n = 0	4	6	9	12	15	24
0	5.49		11.785	12.362	12.672	12.690	12.792	12.740	12.342
3	5.58		11.176	11.996	12.447	12.792	12.771	12.792	12.602
6	5.65		10.991	11.813	12.342	12.785	12.716	12.778	12.716
10	5.78		9.431	10.964	11.785	12.591	12.477	12.613	12.792
